# Supplementary material for: Vaccine hesitancy and HPV vaccine uptake among male and female youth in Switzerland: a cross-sectional study
Source: BMJ Open. 2022 Apr 21;12(4):e053754. doi: 10.1136/bmjopen-2021-053754 (PMC9024257; doi:10.1136/bmjopen-2021-053754)
Supplement: Author's manuscript [file bmjopen-2021-053754.draft_revisions.pdf]

BMJ Open

BMJ Open is committed to open peer review. As part of this commitment we make the peer review history of every article we publish publicly available.

When an article is published we post the peer reviewers' comments and the authors' responses online. We also post the versions of the paper that were used during peer review. These are the versions that the peer review comments apply to.

The versions of the paper that follow are the versions that were submitted during the peer review process. They are not the versions of record or the final published versions. They should not be cited or distributed as the published version of this manuscript.

BMJ Open is an open access journal and the full, final, typeset and author-corrected version of record of the manuscript is available on our site with no access controls, subscription charges or pay-per-view fees (http://bmjopen.bmj.com).

If you have any questions on BMJ Open's open peer review process please email info.bmjopen@bmj.com

BMJ Open

# Vaccine hesitancy and HPV vaccine uptake among male and female youth in Switzerland: a cross-sectional study

| | |
|---|---|
| Journal: | *BMJ Open* |
| Manuscript ID | bmjopen-2021-053754 |
| Article Type: | Original research |
| Date Submitted by the Author: | 29-May-2021 |
| Complete List of Authors: | Kiener, Laura M.; Kantonsspital Baselland Standort Bruderholz; University of Basel, Medicine<br>Schwendener, Corina L.; Kantonsspital Baselland Standort Bruderholz; University of Basel, Medicine<br>Meier, Audrey; Kantonsspital Baselland Standort Bruderholz; University of Basel, Medicine<br>Reber, Noah; Kantonsspital Baselland Standort Bruderholz; University of Basel, Medicine<br>Muggli, Franco; Rekrutierungszentrum Monte Ceneri, Schweizer Armee<br>Gültekin, Nejla; Kompetenzzentrum für Militär- und Katastrophenmedizin, Eidgenössisches Departement für Verteidigung, Bevölkerungsschutz und Sport VBS Schweizer Armee<br>Jafflin, Kristen; Swiss Tropical and Public Health Institute; University of Basel<br>Schärli Maurer, Susanna; Rekrutierungszentrum Aarau, Schweizer Armee<br>Huber, Benedikt; HFR Fribourg Cantonal Hospital, Department of Pediatrics<br>Merten, Sonja; Swiss Tropical and Public Health Institute; University of Basel<br>Deml, Michael; Swiss Tropical and Public Health Institute; University of Basel<br>Tarr, Philip ; Kantonsspital Baselland Standort Bruderholz; University of Basel, Medicine |
| Keywords: | COMPLEMENTARY MEDICINE, PREVENTIVE MEDICINE, PUBLIC HEALTH, SEXUAL MEDICINE, VIROLOGY |
| | |

SCHOLARONE™
Manuscripts

*I, the Submitting Author has the right to grant and does grant on behalf of all authors of the Work (as defined in the below author licence), an exclusive licence and/or a non-exclusive licence for contributions from authors who are: i) UK Crown employees; ii) where BMJ has agreed a CC-BY licence shall apply, and/or iii) in accordance with the terms applicable for US Federal Government officers or employees acting as part of their official duties; on a worldwide, perpetual, irrevocable, royalty-free basis to BMJ Publishing Group Ltd ("BMJ") its licensees and where the relevant Journal is co-owned by BMJ to the co-owners of the Journal, to publish the Work in this journal and any other BMJ products and to exploit all rights, as set out in our licence.*

*The Submitting Author accepts and understands that any supply made under these terms is made by BMJ to the Submitting Author unless you are acting as an employee on behalf of your employer or a postgraduate student of an affiliated institution which is paying any applicable article publishing charge ("APC") for Open Access articles. Where the Submitting Author wishes to make the Work available on an Open Access basis (and intends to pay the relevant APC), the terms of reuse of such Open Access shall be governed by a Creative Commons licence – details of these licences and which Creative Commons licence will apply to this Work are set out in our licence referred to above.*

*Other than as permitted in any relevant BMJ Author's Self Archiving Policies, I confirm this Work has not been accepted for publication elsewhere, is not being considered for publication elsewhere and does not duplicate material already published. I confirm all authors consent to publication of this Work and authorise the granting of this licence.*

For peer review only

# Vaccine hesitancy and HPV vaccine uptake among male and female youth in Switzerland: a cross-sectional study

**Authors**: Laura M. Kiener [1,2] *, Corina L. Schwendener[1,2] * Kristen Jafflin [3,2], Audrey Meier[1,2], Noah Reber[1,2], Susanna Schärli[4], Franco Muggli[5], Nejla Gültekin[6], Benedikt M. Huber[7], Sonja Merten[3,2], Michael J. Deml [3,2], Philip E. Tarr [1,2]

*: equal contribution

**Affiliations:**

1. University Department of Medicine, Kantonsspital Baselland, University of Basel, 4101 Bruderholz, Switzerland
2. University of Basel, Petersplatz 1, 4051 Basel, Switzerland
3. Swiss Tropical and Public Health Institute, Socinstr. 57, 4051 Basel, Switzerland
4. Rekrutierungszentrum Aarau, Schweizer Armee, Aarau, Switzerland
5. Rekrutierungszentrum Monte Ceneri, Schweizer Armee, Monte Ceneri, Switzerland
6. Kompetenzzentrum für Militär- und Katastrophenmedizin, Eidgenössisches Departement für Verteidigung, Bevölkerungsschutz und Sport VBS Schweizer Armee, Ittigen, Switzerland
7. Department of Pediatrics, HFR Fribourg Cantonal Hospital, Fribourg, Switzerland

**Correspondence:** Prof. Dr. med. Philip E. Tarr, National Research Program NFP74 Vaccine Hesitancy, University Dept. of Medicine, Kantonsspital Baselland, University of Basel, 4101 Bruderholz, Switzerland, philip.tarr@unibas.ch, phone +41 (61) 436 2212, fax +41 (61) 436 3670

**Word count**

2854 (exclusive title page, authors' contribution statements, abstract, keywords, strengths and limitations of this study, acknowledgments, ethics approval)

**Key words:**

HPV vaccine, HPV under-immunization, Youth Attitudes about Vaccines Scale, Parental Attitudes about Childhood vaccination, youth, vaccine hesitancy

For peer review only

## Author Contributions

Ms. Kiener and Ms. Schwendener recruited participating youth, conducted interviews, carried out the analysis, drafted the initial manuscript, and reviewed and revised the manuscript.

Dr. Jafflin conceptualized and designed the study, designed the data collection instruments, coordinated and supervised data collection, carried out the analyses, and reviewed and revised the manuscript.

Ms. Meier and Mr. Reber recruited participating youth, conducted interviews, and reviewed and revised the manuscript.

Dr. Schärli, Dr. Muggli and Dr. Gültekin recruited participating youth, and reviewed and revised the manuscript.

Dr. Huber conceptualized and designed the study, recruited participating providers, and reviewed and revised the manuscript.

Dr. Merten conceptualized and designed the study, designed the data collection instruments, coordinated and supervised data collection, and reviewed and revised the manuscript.

Dr. Deml conceptualized and designed the study, designed the data collection instruments, and reviewed and revised the manuscript.

Dr. Tarr conceptualized and designed the study, designed the data collection instruments, recruited participating providers, coordinated and supervised data collection, carried out the analyses, and reviewed and revised the manuscript.

All authors approved the final manuscript as submitted and agree to be accountable for all aspects of the work. All authors attest they meet the ICMJE criteria for authorship.

For peer review only

## Abstract

**Objectives** Identifying factors associated with HPV vaccine uptake is essential for designing successful vaccination programs. We aimed to examine the association between vaccine hesitancy (VH) and HPV vaccine uptake among male and female youth in Switzerland.

**Design** With a cross-sectional study, an interview-based questionnaire was used to collect information on sociodemographic factors, vaccination records, and to measure the prevalence of VH using the Youth Attitudes about Vaccines scale (YAV-5), a modified version of the Parent Attitudes about Childhood Vaccinations (PACV) survey instrument.

**Setting and Participants** Eligible male and female participants, 15-26 years of age, were recruited through physicians' offices and military enlistment in all three language regions of Switzerland. Of 1001 participants, we included 674 participants with a vaccination record available (415 males, 259 females) in this study.

**Primary and secondary outcome measures** The outcome was uptake for HPV vaccine (having received $\geq$1 dose of HPV vaccine). Co-variates were VH, sex, age and other sociodemographics.

**Results** 151 (58%) female and 64 (15%) male participants received $\geq$1 dose of HPV vaccine. 81 (31%) female and 92 (22%) male participants were VH (YAV-5-Score $\geq$50). The odds for being unvaccinated were higher for VH females than non-VH females, adjusted odds ratio (OR)=4.90 (95% CI, 2.53-9.50), but similar among VH and non-VH males, OR=1.90 (95% CI, 0.84-4.31). The odds for being unvaccinated were lower for younger males (born on or after July 1, 2002) than older males (born before July 1, 2002), OR=0.34 (95% CI, 0.14-0.81), but we found no association between age and vaccine uptake for female youth, OR=0.97 (95% CI, 0.48-1.97).

**Conclusions** VH was associated with lower HPV vaccine uptake in female youth but not male youth in our study sample in Switzerland. Our findings suggest that issues other than VH contribute to HPV under-immunization in male youth in Switzerland.

For peer review only

## Strengths and limitations of this study:

- This study is the first using the YAV-5 to measure VH in youth and to associate VH with HPV vaccination uptake.

- The sample includes females and a large number of male youth from all three language regions of Switzerland, which allowed us to gain important insight into males' perspectives and vaccine uptake.

- The findings of this study are likely comparable to other high-income countries in which HPV vaccination programs have recently been extended to young males and where HPV vaccine is covered by health insurance or state-funded programs.

- The large age range of the participants might diminish effects of HPV vaccine recommendations and programs since programs have changed frequently in the past years.

- As our participant sampling is not representative, VH participants might be less likely to participate in a study examining VH than non-VH persons.

For peer review only

# 1. Introduction

Identifying factors contributing to under-immunization is essential for designing successful human papillomavirus (HPV) vaccination programs. Previous studies have shown various influences on HPV under-immunization in female youth, including access barriers, lack of efficient school vaccination programs, and concerns about safety and side effects [1-9]. Because many countries have only recently recommended HPV vaccine for males, information on the determinants of HPV vaccine uptake in men is limited, with lack of awareness and knowledge, access barriers, and lack of health care provider recommendation being previously reported determinants [4-6, 10-17].

Vaccine hesitancy (VH), which "refers to delay in acceptance or refusal of vaccination despite availability of vaccination services" [18] was identified by the World Health Organization as among 10 threats to global health in 2019 [19]. Parental VH contributes to under-immunization of their children for both childhood vaccines [20-22] and HPV vaccine [23, 24].

Since the determinants of HPV vaccination are context specific, local studies are needed. Additionally, previous studies investigating VH towards HPV vaccine had a limited sample size [24] or only investigated parents' perspectives without taking into account youth perspectives [13, 17, 23, 25-28]. Exploring parents' perspectives is appropriate in the target group for HPV vaccine, i.e. adolescents 11-14 years of age, ideally before starting sexual activity. However, many countries, including Switzerland, allow adolescents as young as 14 to make vaccine decisions [29]. In addition, HPV vaccine is now recommended as a catch-up vaccine until 26 years in many countries, and until age 45 in the US [30]. Therefore, youth and young adult attitudes and perspectives on HPV vaccine, including VH, merit further investigation on a larger scale. This study addresses these research gaps by examining youth's VH in relationship to their HPV vaccination uptake.

The Swiss Federal Office of Public Health (FOPH) and the Federal Vaccination Commission have recommended HPV vaccine as a routine vaccine for girls and young women since 2007



For peer review only - http://bmjopen.bmj.com/site/about/guidelines.xhtml

[31]. In 2015, the FOPH added HPV vaccine to the listed of optional vaccines for boys and young men [32]. HPV vaccine is a key vaccine included in the Swiss National Vaccination Strategy [33], and cantonal (state) vaccination programs fully cover the cost of HPV vaccination for both males and females in the recommended age groups. Nevertheless, HPV vaccination coverage in Switzerland remains low [34, 35]. While vaccine uptake has increased in the last decade [2], in the most recent assessment by the FOPH (period 2017-2019), only 64% and 20% of 16-year old girls and boys, respectively, had received ≥1 dose of HPV vaccine [34]. Regional differences in uptake have been attributed to socio-cultural factors, differences in vaccination policies, and differences in the strategies and efforts of the local health departments [36, 37].

We use the Youth Attitudes about Vaccines Scale (YAV-5), a modified version of the Parent Attitudes about Childhood Vaccination (PACV) tool, originally developed by Opel and colleagues, in order to measure youth VH [38]. The PACV is a validated measure of VH predicting under-immunization in children in the US [20]. However, researchers found no association between PACV score and under-immunization with adolescent vaccines, including HPV, in the US [39]. The objectives of this study are to particularly assess how VH influences HPV vaccine uptake among males and females 15-26 years old in our sample in Switzerland. We hypothesize that (1) a YAV-5-score ≥50 (indicating VH) in youth is associated with lower HPV vaccine uptake, and (2) the association between YAV-5- score and HPV vaccine uptake is stronger among females than among males.



For peer review only - http://bmjopen.bmj.com/site/about/guidelines.xhtml

## 2. Methods

We conducted this cross-sectional study in the context of the Swiss National Research Program NRP74 on the determinants of VH and under-immunization with HPV and childhood vaccines in Switzerland [40]. The local ethics committee (Ethikkommission Nordwest- und Zentralschweiz, EKNZ; project ID number 2017-00725) approved the study, and ethics approval covers all study participants, including adolescent and adult patients, as well as biomedical and CAM providers. All participants provided informed consent after the nature and possible consequences of the study had been fully explained. Full details on the study, including recruitment methods, power calculation, development of the survey and a description of the situation of biomedicine and complementary and alternative (CAM) medicine in Switzerland have been previously published [41].

**2.1 Sampling.** Participants were male and female youth 15-26 years of age. We recruited participants in the offices of physicians providing either conventional biomedicine alone ("biomedical provider") or biomedicine combined with complementary and alternative medicine ("CAM provider"). We recruited participants from January 2019 to May 2020 in urban and rural areas in the three main language regions of Switzerland (German, French and Italian). In order to supplement the low number of male participants, we also recruited study participants during their military enlistment in the Swiss Army. Military service is mandatory for all Swiss men, with enlistment taking place at around 19 years of age. Participants recruited in physicians' offices were interviewed on the phone after the physician visit. Participants recruited through the military were interviewed face-to-face on site. Medical students trained by senior researchers in recruiting and obtaining informed consent conducted the interviews, which lasted 25-35 minutes. Data collectors entered all data into open data kit (ODK) using tablets.

**2.2 YAV-5.** We measure vaccine hesitancy using the Youth attitudes about Vaccines scale (YAV-5), an adapted version of the PACV survey instrument, which was originally developed by Opel and colleagues [38]. We have previously validated a 5-item short version of the



For peer review only - http://bmjopen.bmj.com/site/about/guidelines.xhtml

PACV in German, French, Italian and English regarding childhood vaccines [42]. For this project we adapted the questions for use in youth, and validated the YAV-5 in German, French, Italian and English language [Olarewaju, manuscript in preparation]. The adaptions we made for the YAV-5 instrument involved asking youth about their attitudes about adolescent vaccines. Each YAV-5-item is scored from 0-2. Individual scores are then summed and transformed to a 0-100 scale using simple linear transformation [20, 43]. We will refer to participants with a score <50 as non-VH, and participants with a score $\geq$50 as VH [20, 44].

**2.3 Survey.** In addition to the YAV-5, the survey included items about participants' socio-demographic characteristics such as language and birthdates. After finalizing the survey in English, we translated it into German, French and Italian using the forward and backward method [41], pre-tested, adjusted and piloted the survey as previously described [42]. We determine youths' HPV vaccination status through an examination of copies of their vaccination record and, for those not having a vaccination record because of they haven't received any vaccines, a personal statement to this effect. Being unvaccinated was defined as not having received $\geq$1 dose of HPV vaccine at the time of the interview.

**2.4 Participant age groups**. We divided participants into 2 age groups based on the later introduction of the recommendation of the HPV vaccine for boys in Switzerland. HPV vaccine has been covered by health insurance for male adolescents 11 to 14 years of age and as a catch-up vaccination until the age of 26 years since July 1, 2016. We therefore refer to male participants who were 14 years of age or older when insurance coverage for HPV vaccination began (born before July 1, 2002) as "older" and those who were under 14 at this time (born on or after July 1, 2002) as "younger" participants, respectively. For comparison, we applied the same age cut-offs to female participants.

**2.6 Patient and public involvement**

This study did not include patient or public involvement in designing the study, commenting the outcomes, interpreting the results or reviewing this manuscript.



For peer review only - http://bmjopen.bmj.com/site/about/guidelines.xhtml

**2.7 Quantitative data analysis.** We present descriptive statistics to summarize participants' age, place of birth, language region, YAV-5 score, and their HPV vaccination status. We calculated unadjusted odds ratios for being unvaccinated for all independent variables. We then conducted multi-level logistic regressions with random effects by recruitment setting (military enlistment, biomedical office, CAM office) to calculate adjusted odds ratios. Control variables included younger vs. older age group, being Swiss born, language region, and living in an area with local HPV vaccine school programs (Model 0). Next, we extended the model by adding the YAV-5-score as a new control variable for testing our hypotheses (Model 1). We also stratified analyses by sex. We analyzed all data using STATA (v12.0, Stata Corp, College Station, TX).

For peer review only

## 3. Results

**3.1 Participants**. We completed interviews with 1001 youth. For our analysis, we exclude youth who did not match our aging criteria (n=15), who did not provide a correct postcode (n=10), or who did not provide vaccination records for any reason other than not having one due to not vaccinating (n=302).

All analyses are therefore based on 674 participants, of which 415 (62%) are male and 259 (38%) are female. Their characteristics are shown in **Table 1**. The majority of participants were Swiss born, spoke German and lived in the German-speaking region of Switzerland, consistent with this being the largest Swiss language region. We recruited the majority of male participants during military enlistment and the majority of female participants in biomedical physicians' offices. We recruited more female than male participants in CAM physicians' offices. 151 (58%) female and 64 (15%) male participants had received ≥1 dose of HPV vaccine. 81 (31%) females and 92 (22%) males were VH with a YAV-5-score ≥50.

**3.2. Determinants of HPV vaccine uptake, entire study population**. In univariable analysis, the odds of being unvaccinated with HPV were higher for males than for females (odds ratio [OR]=5.28; 95% confidence interval [CI], 3.17-8.80), and higher among participants recruited during military enlistment, most of whom were male, and in CAM offices (**Table 2**). VH youth had higher odds of being unvaccinated than non-VH youth, OR=2.75 (95% CI, 1.69-4.48). We found no significant difference in the odds of being unvaccinated in the univariable analysis based on age, being Swiss-born, language region or living in areas with local school vaccination programs. In the multivariable model 0, the odds were similar to the odds calculated in univariable analysis, with the exception of Swiss-born participants, whose odds for being unvaccinated were higher than foreign-born participants' (**Table 2**). In the multivariable model 1, after adding VH, the odds remained essentially unchanged: as in univariable analysis, the odds of being unvaccinated were higher among VH participants than non-VH participants, OR=3.20 (95% CI, 1.94-5.26).

For peer review only

**3.3. Determinants of HPV vaccine uptake, stratified by sex**. The univariable odds for being unvaccinated were lower for younger males compared to older males, but we found no significant difference in the odds among younger and older females (**Table 3**). The univariable odds for being unvaccinated were higher for females recruited in CAM offices than for females recruited in biomedical offices, and the odds for being unvaccinated were higher in VH females than non-VH females, OR=5.29 (95% CI, 2.71-10.33). We found no correlation between recruitment setting or VH and vaccine uptake for males (**Table 3**). There was no significant association between place of birth, language region, and school vaccination programs and vaccine uptake. In the multivariable model 0 and model 1, results for both males and females were similar to univariable results (**Table 3**). The odds for being unvaccinated were higher among VH females than non-VH females, OR=4.90 (95% CI, 2.53-9.50), but we found no association between VH and vaccine uptake for males, OR=1.90 (95% CI, 0.84-4.31). Similar, the odds for being unvaccinated were higher among females recruited in a CAM office than females recruited in a biomedical office even when controlling for VH in model 1, OR=2.52 (95%CI, 1.13-5.63), but for males we found no association between recruitment setting and vaccine uptake, OR=1.43 (95% CI, 0.39-5.20).

For peer review only

## 4. Discussion

This study shows that male youth have higher odds of being unvaccinated against HPV than females, despite fewer males being VH in our study. We found a significant association between VH and HPV under-immunization only for females. The main finding of the present study shows that VH is a major determinant of HPV under-immunization for female youth but not for male.

HPV vaccine uptake in our study was far lower in males than in females, despite the HPV vaccine recommendation having been officially made >4 years prior to the interviews. Our finding of higher HPV vaccine uptake in younger male participants than older ones suggests that the Swiss HPV vaccine recommendation for boys in 2015 led to a higher HPV vaccine uptake among younger eligible males.

The VH prevalence in our study was almost 10 percentage points lower in males than in females, but this difference might be influenced by the fact that female participants were selected in a less random sample compared to males, who mostly were recruited through military enlistment. Still, this finding is consistent with previous reports suggesting that in general women tend to be more VH than men [45]. However, as suggested by the WHO's SAGE (Strategic Advisory Group of Experts on Immunization) working group on VH, a high level of VH leads to a lower vaccine demand, but lower VH levels do not necessarily lead to higher vaccine demand [18].

The finding that VH is not significantly associated with HPV under-immunization in males suggests that male youth face additional barriers to HPV vaccination, which may include limited HPV vaccine awareness and knowledge, access barriers, and a lack of priority given to male HPV vaccination by public health authorities and providers. An explanation for higher access to the HPV vaccine among female youth is that catch-up HPV vaccination in young Swiss women is partially done by gynecologists [35]. In contrast, young men in good health see a medical provider less often than young women in good health [46] and may therefore

For peer review only

not receive an HPV vaccine recommendation from any provider [4, 5], nor be aware of the benefits of HPV vaccine [4, 5, 11-14].

To our knowledge, this is the first study that measures VH in youth using the YAV-5 instrument, an adapted version of the PACV survey, in order to examine associations between VH and HPV vaccine uptake. Similarly, we here show a significant association of youth VH and HPV under-immunization in female youth using the YAV-5-score. Our results echo the findings of Preston and colleagues who identified an association between negative attitudes toward HPV vaccine and HPV under-immunization among female, but not among male college students in Florida, USA [24]. We also found that females recruited in the offices of CAM providers have higher odds of being unvaccinated with HPV vaccine, even when controlling for youth vaccine hesitancy. This is consistent with previous research showing that CAM usage is associated with under-immunization [47, 48].

For peer review only

## 5. Strengths and limitations

Strengths of our research include being the first study using the YAV-5 to measure VH in youth and to associate VH with HPV vaccination uptake. Additional strengths of our study include integration of a large number of male youth from all three language regions of Switzerland, which allowed us to gain important insight into males' perspectives and vaccine uptake. Little research has been done on males' HPV vaccine attitudes and uptake, particularly in the Swiss setting.

Our findings are likely comparable to other high-income countries in which HPV vaccination programs have recently been extended to young males and where HPV vaccine is covered by health insurance. Since the YAV-5 survey instrument has been previously validated to measure VH in youth [Olarewaju, manuscript in preparation], our study results can lead to meaningful comparisons with other populations.

Our study also has limitations. There was no evidence in our study that the availability of an HPV school vaccination program was associated with increased HPV vaccination uptake. This stands in contrast to a 2018 Swiss study on the association between school vaccination programs and HPV vaccine uptake in young women [2]. However, our study has important limitations for investigating the impact of school programs due to the large age range of participants, a non-representative sample, lack of data on schooling received, and the possibility that youth no longer live in the same area as when they would have received such schooling.

Our sample is not representative for Switzerland. Additional potential sources of bias result from us not being able to include participants who do not go to a physicians' office. Finally, VH persons might be less likely to participate in a study examining VH than non-VH persons. Future research could address these gaps by using a random sampling approach throughout the population with the aims of gathering representative data.



For peer review only - http://bmjopen.bmj.com/site/about/guidelines.xhtml

## 6. Conclusion

By administering the YAV-5, a validated instrument for measuring VH in youth, to a large sample of youth in Switzerland, our results suggest that VH may be a major determinant of HPV under-immunization for female youth. While male youth had higher odds of being unvaccinated, VH is not associated with HPV under-immunization in male youth. This suggests that issues other than VH, including HPV vaccine awareness and knowledge and access to HPV vaccination for youth who do not have regular contacts with the healthcare system, may still be predominant reasons for low vaccine uptake among male youth in Switzerland and should be explored in further research. Furthermore, improved access to HPV vaccination seems a key issue for the many eligible youth who do not have regular contacts with the healthcare system.

## Acknowledgements

We would like to thank the participating adolescents, young adults and physicians and their medical assistants for their time and effort.

## Disclosure of Potential Conflict of Interest

All authors: no conflicts.

## Funding

The study is funded via the Swiss National Science Foundation (Grant Number 407440_167398, recipient: PET), in the setting of National Research Programme (NRP) 74. The study benefited from supplementary postdoctoral fellowship funding from the Nora van Meeuwen-Haefliger-Foundation. No funding was obtained from vaccine manufacturers or the Swiss Federal Office of Public Health.

## Ethics approval

For peer review only

The local ethics committee (Ethikkommission Nordwest- und Zentralschweiz, EKNZ; project ID number 2017-00725) approved the study, and ethics approval covers all study participants, including adolescent and adult patients, as well as biomedical and CAM providers. All participants provided informed consent after the nature and possible consequences of the study had been fully explained.

## Data sharing statement

Data are available upon reasonable request to the corresponding author.

## References


1. Lang, P., et al., *Evolution of the HPV vaccination coverage in Switzerland, 2008-2016*, in *Swiss public health conference*. Basel, 22-23 Nov 2017. [30.03.2021]; Available from: https://organizers-congress.org/frontend/index.php?page_id=4147&additions_conferenceschedule_action=detail&additions_conferenceschedule_controller=paperList&pid=4441&hash=925078cf906fe3575968a0d451fd6a1e106903fb18506bfe44d7fe13e74b74b1.
2. Riesen, M., et al., *Exploring variation in human papillomavirus vaccination uptake in Switzerland: a multilevel spatial analysis of a national vaccination coverage survey.* BMJ Open, 2018. **8**(5): p. e021006.
3. Wymann, M.N., et al., *Human papillomavirus vaccine uptake in adolescence and adherence to cervical cancer screening in Switzerland: a national cross-sectional survey.* Int J Public Health, 2018. **63**(1): p. 105-114.
4. Thompson, E.L., et al., *Human Papillomavirus Vaccination: What Are the Reasons for Nonvaccination Among U.S. Adolescents?* J Adolesc Health, 2017. **61**(3): p. 288-293.
5. Gilkey, M.B., et al., *Do correlates of HPV vaccine initiation differ between adolescent boys and girls?* Vaccine, 2012. **30**(41): p. 5928-34.
6. Rodriguez, S.A., et al., *Factors associated with adolescent HPV vaccination in the U.S.: A systematic review of reviews and multilevel framework to inform intervention development.* Prev Med, 2020. **131**: p. 105968.
7. Canfell, K., et al., *Factors related to vaccine uptake by young adult women in the catch-up phase of the National HPV Vaccination Program in Australia: Results from an observational study.* Vaccine, 2015. **33**(20): p. 2387-94.
8. Amadane, M., et al., *Characteristics of HPV-unvaccinated undergraduate health students in Switzerland, a cross sectional study.* Arch Public Health, 2019. **77**: p. 29.
9. Barrense-Dias Y., et al., *Sexual health and behavior of young people in Switzerland.* Raison de santeé, 2018. **291**.
10. Sherman, S.M. and E. Nailer, *Attitudes towards and knowledge about Human Papillomavirus (HPV) and the HPV vaccination in parents of teenage boys in the UK.* PLoS One, 2018. **13**(4): p. e0195801.
11. Cooper, D.L., et al., *Examining HPV Awareness, Sexual Behavior, and Intent to Receive the HPV Vaccine Among Racial/Ethnic Male College Students 18-27 years.* Am J Mens Health, 2018. **12**(6): p. 1966-1975.
12. Perez, S., et al., *Untangling the psychosocial predictors of HPV vaccination decision-making among parents of boys.* Vaccine, 2017. **35**(36): p. 4713-4721.


For peer review only

13. Lacombe-Duncan, A., P.A. Newman, and P. Baiden, *Human papillomavirus vaccine acceptability and decision-making among adolescent boys and parents: A meta-ethnography of qualitative studies.* Vaccine, 2018. **36**(19): p. 2545-2558.
14. Kasymova, S., *Human papillomavirus (HPV) and HPV vaccine knowledge, the intention to vaccinate, and HPV vaccination uptake among male college students.* J Am Coll Health, 2020: p. 1-15.
15. Voss, D.S. and L.G. Wofford, *Human Papillomavirus Vaccine Uptake in Adolescent Boys: An Evidence Review.* Worldviews Evid Based Nurs, 2016. **13**(5): p. 390-395.
16. Dibble, K.E., et al., *A Systematic Literature Review of HPV Vaccination Barriers Among Adolescent and Young Adult Males.* J Adolesc Young Adult Oncol, 2019. **8**(5): p. 495-511.
17. Radisic, G., et al., *Factors associated with parents' attitudes to the HPV vaccination of their adolescent sons : A systematic review.* Prev Med, 2017. **95**: p. 26-37.
18. MacDonald, N.E., *Vaccine hesitancy: Definition, scope and determinants.* Vaccine, 2015. **33**(34): p. 4161-4.
19. World health organization. *Ten threats to global health in 2019.* [11.02.2021]; Available from: https://www.who.int/news-room/spotlight/ten-threats-to-global-health-in-2019.
20. Opel, D.J., et al., *The relationship between parent attitudes about childhood vaccines survey scores and future child immunization status: a validation study.* JAMA Pediatr, 2013. **167**(11): p. 1065-71.
21. Williams, S.E., et al., *Screening Tool Predicts Future Underimmunization Among a Pediatric Practice in Tennessee.* Clin Pediatr (Phila), 2016. **55**(6): p. 537-42.
22. Napolitano, F., A. D'Alessandro, and I.F. Angelillo, *Investigating Italian parents' vaccine hesitancy: A cross-sectional survey.* Hum Vaccin Immunother, 2018. **14**(7): p. 1558-1565.
23. Szilagyi, P.G., et al., *Prevalence and characteristics of HPV vaccine hesitancy among parents of adolescents across the US.* Vaccine, 2020. **38**(38): p. 6027-6037.
24. Preston, S.M. and W.W. Darrow, *Are Men Being Left Behind (Or Catching Up)? Differences in HPV Awareness, Knowledge, and Attitudes Between Diverse College Men and Women.* Am J Mens Health, 2019. **13**(6): p. 1557988319883776.
25. Della Polla, G., et al., *HPV vaccine hesitancy among parents in Italy: a cross-sectional study.* Hum Vaccin Immunother, 2020. **16**(11): p. 2744-2751.
26. Sherman, S.M., et al., *Knowledge, attitudes and awareness of the human papillomavirus among health professionals in New Zealand.* PLoS One, 2018. **13**(12): p. e0197648.
27. Perez, S., et al., *Psychosocial determinants of parental human papillomavirus (HPV) vaccine decision-making for sons: Methodological challenges and initial results of a pan-Canadian longitudinal study.* BMC Public Health, 2016. **16**(1): p. 1223.
28. Newman, P.A., et al., *Parents' uptake of human papillomavirus vaccines for their children: a systematic review and meta-analysis of observational studies.* BMJ Open, 2018. **8**(4): p. e019206.
29. Yang, Y.T., R.S. Olick, and J. Shaw, *Adolescent Consent to Vaccination in the Age of Vaccine-Hesitant Parents.* JAMA Pediatr, 2019.
30. Meites, E., et al., *Human Papillomavirus Vaccination for Adults: Updated Recommendations of the Advisory Committee on Immunization Practices.* MMWR Morb Mortal Wkly Rep, 2019. **68**(32): p. 698-702.
31. Federal office of public health, *Recommandations de vaccination contre les papillomavirus humains (HPV)*, D.e. recommandations, Editor. 2008. Document in French: https://www.bag.admin.ch/dam/bag/fr/dokumente/mt/i-und-b/richtlinien-empfehlungen/empfehlungen-spezifische-erreger-krankheiten/hpv/hpv-humane-papillomaviren-empfehlung-impfung.pdf.download.pdf/ofsp-recommandations-hpv.pdf
32. Federal office of public health, *Vaccination contre les HPV : recommandation de vaccination complémentaire pour les garçons et jeunes hommes âgés de 11 à 26 ans.* BAG Bulletin, 2015. **10**: p. 141-150. Document in French: https://www.bag.admin.ch/dam/bag/fr/dokumente/mt/i-und-b/richtlinien-


For peer review only

empfehlungen/empfehlungen-spezifische-erreger-krankheiten/hpv/hpv-impfung-jungen-maenner.pdf.download.pdf/hpv-vaccination-garcons.pdf
33. Federal office of public health, *Stratégie nationale de vaccination*. 2017. Document in French: https://www.bag.admin.ch/dam/bag/fr/dokumente/mt/i-und-i/nsi/nsi-strategie-vollversion.pdf.download.pdf/nsi-strategie-vollversion-fr.pdf
34. Federal office of public health. *Couverture vaccinale des enfants âgés de 2, 8 et 16 ans en Suisse, 1999-2019*. 2020 30.07.2020 [15.10.2020]; Document in French: https://www.bag.admin.ch/dam/bag/fr/dokumente/mt/i-und-b/durchimpfung/tabelle-durchimpfung.xlsx.download.xlsx/tabelle-durchimpfung-200730-fr.xlsx.
35. Federal office of public health, *La vaccination contre le HPV en Suisse: résultats d'une enquête nationale réalisée en 2014.* BAG Bulletin, 2015. **23**: p. 445-452. Document in French: https://www.bag.admin.ch/dam/bag/fr/dokumente/cc/Kampagnen/Bulletin/2015/BU_15_15_f.pdf.download.pdf/BU_15_15_f.pdf
36. Masserey Spicher, V. and M.G. Weiss, *Policy and socio-cultural differences between cantons in Switzerland with high and low adolescent vaccination coverage for hepatitis B and HPV.* Vaccine, 2019. **37**(52): p. 7539-7546.
37. Riesen, M., et al., *Modeling the consequences of regional heterogeneity in human papillomavirus (HPV) vaccination uptake on transmission in Switzerland.* Vaccine, 2017. **35**(52): p. 7312-7321.
38. Opel, D.J., et al., *Development of a survey to identify vaccine-hesitant parents: the parent attitudes about childhood vaccines survey.* Hum Vaccin, 2011. **7**(4): p. 419-25.
39. Roberts, J.R., et al., *Vaccine hesitancy among parents of adolescents and its association with vaccine uptake.* Vaccine, 2015. **33**(14): p. 1748-55.
40. National Research Programme 74. *28 Vaccine-sceptical patients and doctors in Switzerland*. 14.02.2021]; Available from: www.nfp74.ch/en/projects/out-patient-care/project-tarr.
41. Deml, M.J., et al., *Determinants of vaccine hesitancy in Switzerland: study protocol of a mixed-methods national research programme.* BMJ Open, 2019. **9**(11): p. e032218.
42. Olarewaju, V.O., et al., *Application of the Parent Attitudes about Childhood Vaccines (PACV) survey in three national languages in Switzerland: Exploratory factor analysis and Mokken scale analysis.* Hum Vaccin Immunother, 2021: p. 1-9.
43. Oladejo, O., et al., *Comparative analysis of the Parent Attitudes about Childhood Vaccines (PACV) short scale and the five categories of vaccine acceptance identified by Gust et al.* Vaccine, 2016. **34**(41): p. 4964-4968.
44. Opel, D.J., et al., *Washington State Licensed Child Care Facility Directors' Perspectives on Childhood Immunization.* J Pediatric Infect Dis Soc, 2013. **2**(1): p. 40-9.
45. Baumgaertner, B., et al., *Risk of disease and willingness to vaccinate in the United States: A population-based survey.* PLoS Med, 2020. **17**(10): p. e1003354.
46. Ashman, J.J., P. Rui, and T. Okeyode, *Characteristics of Office-based Physician Visits, 2016.* NCHS Data Brief, 2019(331): p. 1-8.
47. Zuzak, T.J., et al., *Attitudes towards vaccination: users of complementary and alternative medicine versus non-users.* Swiss Med Wkly, 2008. **138**(47-48): p. 713-8.
48. Attwell, K., et al., *"Do-it-yourself": Vaccine rejection and complementary and alternative medicine (CAM).* Soc Sci Med, 2018. **196**: p. 106-114.

For peer review only

**Table 1**. Participant characteristics for full sample and by sex

| | Total (n=674) n (%) | | Male (n=415) n (%) | | Female (n=259) n (%) | |
|---|---|---|---|---|---|---|
| **Age in years (median, IQR)** | 19.1 | (18.1, 20.7) | 19.2 | (18.4, 20.1) | 19.0 | (16.8, 21.7) |
| Born before July 1, 2002 | 563 | (83.5) | 370 | (89.2) | 193 | (74.5) |
| Born on or after July 1, 2002 | 111 | (16.5) | 45 | (10.8) | 66 | (25.5) |
| Swissborn | 621 | (92.1) | 382 | (92.1) | 239 | (92.3) |
| **Language region** | | | | | | |
| German | 494 | (73.3) | 321 | (77.4) | 173 | (66.8) |
| Italian | 137 | (6.4) | 78 | (18.8) | 59 | (22.8) |
| French | 43 | (20.3) | 16 | (3.9) | 27 | (10.4) |
| **Participant recruitment Setting** | | | | | | |
| Military enlistment | 287 | (42.6) | 283 | (68.2) | 4 | (1.5) |
| Biomedical provider | 280 | (41.5) | 97 | (23.4) | 183 | (70.7) |
| CAM provider | 107 | (15.9) | 35 | (8.4) | 72 | (27.8) |
| **HPV vaccine school program in area of their residence** | | | | | | |
| School program available | 366 | (54.3) | 232 | (55.9) | 134 | (51.7) |
| **Vaccination status** | | | | | | |
| Has received $\geq$ 1 dose of HPV vaccine | 215 | (31.9) | 64 | (15.4) | 151 | (58.3) |
| **YAV-5-Score** | | | | | | |
| Score $\geq$50 (hesitant) | 173 | (25.7) | 92 | (22.2) | 81 | (31.3) |
| YAV-5-Score mean (SD) | 32.3 | (23.4) | 30.4 | (22.3) | 35.3 | (24.8) |
| YAV-5-Score median (IQR) | 30.0 | (20, 50) | 30.0 | (10, 40) | 30.0 | (20, 50) |

Note: CAM, Complementary and alternative medicine; IQR, interquartile range; SD, standard deviation; YAV-5, Youth Attitudes about Vaccines Scale.

For peer review only



For peer review only - http://bmjopen.bmj.com/site/about/guidelines.xhtml

**Table 2**. Unadjusted and adjusted odds of not having received ≥1 dose of HPV vaccine, controlling for sex, age groups, place of birth, language region, recruitment setting, availability of HPV vaccine school program in the area of their residence, and VH.

| | **Univariable Analysis** | | | **Model 0** | | | **Model 1** | | |
|---|---|---|---|---|---|---|---|---|---|
| | OR | CI (95%) | | AOR | CI (95%) | | AOR | CI (95%) | |
| **Sex** | | | | | | | | | |
| Female | (reference) | | | (reference) | | | (reference) | | |
| Male | **5.28** | **(3.17** | **8.80)** | **5.16** | **(3.06** | **8.70)** | **5.77** | **(3.40** | **9.79)** |
| **Age groups based on birthdates** | | | | | | | | | |
| Born before July 1, 2002 | (reference) | | | (reference) | | | (reference) | | |
| Born on or after July 1, 2002 | 0.74 | (0.44 | 1.26) | 0.72 | (0.42 | 1.21) | 0.70 | (0.41 | 1.19) |
| **Place of birth** | | | | | | | | | |
| Outside Switzerland | (reference) | | | (reference) | | | (reference) | | |
| Switzerland | 1.85 | (0.95 | 3.60) | **2.03** | **(1.02** | **4.06)** | **2.05** | **(1.02** | **4.10)** |
| **Language region** | | | | | | | | | |
| German | (reference) | | | (reference) | | | (reference) | | |
| French | 0.79 | (0.33 | 1.89) | 0.89 | (0.39 | 2.04) | 0.87 | (0.38 | 1.98) |
| Italian | 0.54 | (0.26 | 1.12) | 0.57 | (0.29 | 1.13) | 0.60 | (0.31 | 1.17) |
| **Participant recruitment setting** | | | | | | | | | |
| Biomedical | (reference) | | | (reference) | | | (reference) | | |
| CAM | **2.46** | **(1.33** | **4.55)** | **2.29** | **(1.21** | **4.32)** | **2.09** | **(1.13** | **3.89)** |
| Military enlistment | **7.29** | **(2.64** | **20.09)** | 2.60 | (0.99 | 6.80) | **2.79** | **(1.15** | **6.79)** |
| **HPV vaccine school program in area of their residence** | | | | | | | | | |
| Not available | (reference) | | | (reference) | | | (reference) | | |
| Available | 1.37 | (0.85 | 2.21) | 1.26 | (0.72 | 2.19) | 1.20 | (0.69 | 2.08) |
| **YAV-5-Score** | | | | | | | | | |
| Score < 50 (not hesitant) | (reference) | | | | | | (reference) | | |
| Score ≥ 50 (hesitant) | **2.75** | **(1.69** | **4.48)** | | | | **3.20** | **(1.94** | **5.26)** |

Note: AOR, adjusted odds ratio; CAM, complementary and alternative medicine; CI, confidential interval; OR, odds ratio; YAV-5, Youth Attitudes about Vaccines Scale.



For peer review only - http://bmjopen.bmj.com/site/about/guidelines.xhtml

**Table 3**. Unadjusted and adjusted odds of having received ≥1 dose of HPV vaccine stratified by sex; controlling for participant sex, age groups, place of birth, language region, recruitment setting, availability of HPV vaccine school program in the area of their residence and VH.

| | **Univariable Analysis, Male Only (n=415)** | | **Model 0, male only (n= 415)** | | **Model 1, male only (n=415)** | | **Univariable Analysis, Female Only (n=259)** | | **Model 0, female only (n=259)** | | **Model 1, female only (n=259)** | |
|---|---|---|---|---|---|---|---|---|---|---|---|---|
| | OR | CI (95%) | AOR | CI (95%) | AOR | CI (95%) | OR | CI (95%) | AOR | CI (95%) | AOR | CI (95%) |
| **Place of birth** | | | | | | | | | | | | |
| Outside Switzerland | (reference) | | (reference) | | (reference) | | (reference) | | (reference) | | (reference) | |
| Switzerland | 2.10 | (0.84 5.23) | 1.77 | (0.69 4.51) | 1.71 | (0.67 4.34) | 2.14 | (0.66 6.95) | 2.10 | (0.64 6.86) | 2.38 | (0.68 8.32) |
| **Age groups based on birthdates** | | | | | | | | | | | | |
| Born before July 1, 2002 | (reference) | | (reference) | | (reference) | | (reference) | | (reference) | | (reference) | |
| Born on or after July 1, 2002 | **0.29** | **(0.12 0.71)** | **0.34** | **(0.14 0.81)** | **0.31** | **(0.13 0.73)** | 0.94 | (0.46 1.93) | 0.97 | (0.48 1.97) | 1.14 | (0.56 2.33) |
| **Language region** | | | | | | | | | | | | |
| German | (reference) | | (reference) | | (reference) | | (reference) | | (reference) | | (reference) | |
| French | 0.50 | (0.11 2.33) | 0.72 | (0.16 3.20) | 0.69 | (0.16 3.03) | 0.88 | (0.31 2.52) | 0.99 | (0.34 2.90) | 0.94 | (0.32 2.80) |
| Italian | 0.38 | (0.12 1.21) | 0.57 | (0.18 1.82) | 0.57 | (0.18 1.80) | 0.44 | (0.17 1.12) | 0.56 | (0.20 1.58) | 0.59 | (0.22 1.62) |
| **Participant recruitment setting** | | | | | | | | | | | | |
| Biomedical | (reference) | | (reference) | | (reference) | | (reference) | | (reference) | | (reference) | |
| CAM | 2.63 | (0.66 10.45) | 1.59 | (0.44 5.82) | 1.43 | (0.39 5.20) | **3.08** | **(1.33 7.14)** | **2.75** | **(1.19 6.33)** | **2.52** | **(1.13 5.63)** |
| Military enlistment | 2.49 | (0.44 13.99) | 1.83 | (0.52 6.47) | 1.84 | (0.57 5.95) | 0.58 | (0.03 11.81) | 0.55 | (0.03 9.53) | 0.59 | (0.03 10.29) |
| **HPV vaccine school program in area of their residence** | | | | | | | | | | | | |
| Not available | (reference) | | (reference) | | (reference) | | (reference) | | (reference) | | (reference) | |
| Available | 1.39 | (0.67 2.91) | 1.21 | (0.53 2.76) | 1.20 | (0.53 2.72) | 1.74 | (0.88 3.43) | 1.14 | (0.48 2.71) | 1.00 | (0.42 2.37) |
| **YAV-5-Score** | | | | | | | | | | | | |
| Score < 50 (not hesitant) | (reference) | | | | (reference) | | (reference) | | | | (reference) | |
| Score ≥ 50 (hesitant) | 1.68 | (0.75 3.76) | | | 1.90 | (0.84 4.31) | **5.29** | **(2.71 10.33)** | | | **4.90** | **(2.53 9.50)** |

Note: AOR, adjusted odds ratio; CAM, complementary and alternative medicine; CI, confidential interval; OR, odds ratio; VH, vaccine hesitant; YAV-5, Youth Attitudes about Vaccines Scale.

For peer review only

**STROBE 2007 (v4) checklist of items to be included in reports of observational studies in epidemiology***

**Checklist for cohort, case-control, and cross-sectional studies (combined)**

| Section/Topic | Item # | Recommendation | Reported on page # |
|---|---|---|---|
| **Title and abstract** | 1 | (*a*) Indicate the study's design with a commonly used term in the title or the abstract | 1 |
| | | (*b*) Provide in the abstract an informative and balanced summary of what was done and what was found | 3 |
| **Introduction** | | | |
| Background/rationale | 2 | Explain the scientific background and rationale for the investigation being reported | 5,6 |
| Objectives | 3 | State specific objectives, including any pre-specified hypotheses | 6 |
| **Methods** | | | |
| Study design | 4 | Present key elements of study design early in the paper | 7 |
| Setting | 5 | Describe the setting, locations, and relevant dates, including periods of recruitment, exposure, follow-up, and data collection | 7 |
| Participants | 6 | (*a*) *Cohort study*—Give the eligibility criteria, and the sources and methods of selection of participants. Describe methods of follow-up<br>*Case-control study*—Give the eligibility criteria, and the sources and methods of case ascertainment and control selection. Give the rationale for the choice of cases and controls<br>*Cross-sectional study*—Give the eligibility criteria, and the sources and methods of selection of participants | 7 |
| | | (*b*) *Cohort study*—For matched studies, give matching criteria and number of exposed and unexposed<br>*Case-control study*—For matched studies, give matching criteria and the number of controls per case | - |
| Variables | 7 | Clearly define all outcomes, exposures, predictors, potential confounders, and effect modifiers. Give diagnostic criteria, if applicable | 7, 8 |
| Data sources/ measurement | 8* | For each variable of interest, give sources of data and details of methods of assessment (measurement). Describe comparability of assessment methods if there is more than one group | 7 |
| Bias | 9 | Describe any efforts to address potential sources of bias | 7 |
| Study size | 10 | Explain how the study size was arrived at | 7 |
| Quantitative variables | 11 | Explain how quantitative variables were handled in the analyses. If applicable, describe which groupings were chosen and why | 7 |
| Statistical methods | 12 | (*a*) Describe all statistical methods, including those used to control for confounding | 8,9 |
| | | (*b*) Describe any methods used to examine subgroups and interactions | 8,9 |
| | | (*c*) Explain how missing data were addressed | 10 |
| | | (*d*) *Cohort study*—If applicable, explain how loss to follow-up was addressed<br>*Case-control study*—If applicable, explain how matching of cases and controls was addressed | - |

For peer review only

| | | | |
|---|---|---|---|
| | | *Cross-sectional study*—If applicable, describe analytical methods taking account of sampling strategy | |
| | | (*e*) Describe any sensitivity analyses | - |
| **Results** | | | |
| Participants | 13* | (a) Report numbers of individuals at each stage of study—eg numbers potentially eligible, examined for eligibility, confirmed eligible, included in the study, completing follow-up, and analysed | 10 |
| | | (b) Give reasons for non-participation at each stage | 10 |
| | | (c) Consider use of a flow diagram | - |
| Descriptive data | 14* | (a) Give characteristics of study participants (eg demographic, clinical, social) and information on exposures and potential confounders | 10, 19 |
| | | (b) Indicate number of participants with missing data for each variable of interest | - |
| | | (c) *Cohort study*—Summarise follow-up time (eg, average and total amount) | - |
| Outcome data | 15* | *Cohort study*—Report numbers of outcome events or summary measures over time | - |
| | | *Case-control study*—Report numbers in each exposure category, or summary measures of exposure | - |
| | | *Cross-sectional study*—Report numbers of outcome events or summary measures | 10, 19 |
| Main results | 16 | (*a*) Give unadjusted estimates and, if applicable, confounder-adjusted estimates and their precision (eg, 95% confidence interval). Make clear which confounders were adjusted for and why they were included | 10, 11, 20, 21 |
| | | (*b*) Report category boundaries when continuous variables were categorized | - |
| | | (*c*) If relevant, consider translating estimates of relative risk into absolute risk for a meaningful time period | - |
| Other analyses | 17 | Report other analyses done—eg analyses of subgroups and interactions, and sensitivity analyses | - |
| **Discussion** | | | |
| Key results | 18 | Summarise key results with reference to study objectives | 12 |
| Limitations | 19 | Discuss limitations of the study, taking into account sources of potential bias or imprecision. Discuss both direction and magnitude of any potential bias | 14 |
| Interpretation | 20 | Give a cautious overall interpretation of results considering objectives, limitations, multiplicity of analyses, results from similar studies, and other relevant evidence | 12, 13, 15 |
| Generalisability | 21 | Discuss the generalisability (external validity) of the study results | 14 |
| **Other information** | | | |
| Funding | 22 | Give the source of funding and the role of the funders for the present study and, if applicable, for the original study on which the present article is based | 15 |

*Give information separately for cases and controls in case-control studies and, if applicable, for exposed and unexposed groups in cohort and cross-sectional studies.
**Note:** An Explanation and Elaboration article discusses each checklist item and gives methodological background and published examples of transparent reporting. The STROBE checklist is best used in conjunction with this article (freely available on the Web sites of PLoS Medicine at http://www.plosmedicine.org/, Annals of Internal Medicine at http://www.annals.org/, and Epidemiology at http://www.epidem.com/). Information on the STROBE Initiative is available at www.strobe-statement.org.

For peer review only

BMJ Open

# Vaccine hesitancy and HPV vaccine uptake among male and female youth in Switzerland: a cross-sectional study

| | |
|---|---|
| Journal: | *BMJ Open* |
| Manuscript ID | bmjopen-2021-053754.R1 |
| Article Type: | Original research |
| Date Submitted by the Author: | 31-Jan-2022 |
| Complete List of Authors: | Kiener, Laura M.; Kantonsspital Baselland Standort Bruderholz; University of Basel, Medicine<br>Schwendener, Corina L.; Kantonsspital Baselland Standort Bruderholz; University of Basel, Medicine<br>Jafflin, Kristen; Swiss Tropical and Public Health Institute; University of Basel<br>Meier, Audrey; Kantonsspital Baselland Standort Bruderholz; University of Basel, Medicine<br>Reber, Noah; Kantonsspital Baselland Standort Bruderholz; University of Basel, Medicine<br>Schärli Maurer, Susanna; Rekrutierungszentrum Aarau, Schweizer Armee<br>Muggli, Franco; Rekrutierungszentrum Monte Ceneri, Schweizer Armee<br>Gültekin, Nejla; Kompetenzzentrum für Militär- und Katastrophenmedizin, Eidgenössisches Departement für Verteidigung, Bevölkerungsschutz und Sport VBS Schweizer Armee<br>Huber, Benedikt; HFR Fribourg Cantonal Hospital, Department of Pediatrics<br>Merten, Sonja; Swiss Tropical and Public Health Institute; University of Basel<br>Deml, Michael; Swiss Tropical and Public Health Institute; University of Basel<br>Tarr, Philip ; Kantonsspital Baselland Standort Bruderholz; University of Basel, Medicine |
| <b>Primary Subject Heading</b>: | Sexual health |
| Secondary Subject Heading: | Complementary medicine, Health policy, Sociology |
| Keywords: | COMPLEMENTARY MEDICINE, PREVENTIVE MEDICINE, PUBLIC HEALTH, SEXUAL MEDICINE, VIROLOGY |
| | |

SCHOLARONE™
Manuscripts

*I, the Submitting Author has the right to grant and does grant on behalf of all authors of the Work (as defined in the below author licence), an exclusive licence and/or a non-exclusive licence for contributions from authors who are: i) UK Crown employees; ii) where BMJ has agreed a CC-BY licence shall apply, and/or iii) in accordance with the terms applicable for US Federal Government officers or employees acting as part of their official duties; on a worldwide, perpetual, irrevocable, royalty-free basis to BMJ Publishing Group Ltd ("BMJ") its licensees and where the relevant Journal is co-owned by BMJ to the co-owners of the Journal, to publish the Work in this journal and any other BMJ products and to exploit all rights, as set out in our licence.*

*The Submitting Author accepts and understands that any supply made under these terms is made by BMJ to the Submitting Author unless you are acting as an employee on behalf of your employer or a postgraduate student of an affiliated institution which is paying any applicable article publishing charge ("APC") for Open Access articles. Where the Submitting Author wishes to make the Work available on an Open Access basis (and intends to pay the relevant APC), the terms of reuse of such Open Access shall be governed by a Creative Commons licence – details of these licences and which Creative Commons licence will apply to this Work are set out in our licence referred to above.*

*Other than as permitted in any relevant BMJ Author's Self Archiving Policies, I confirm this Work has not been accepted for publication elsewhere, is not being considered for publication elsewhere and does not duplicate material already published. I confirm all authors consent to publication of this Work and authorise the granting of this licence.*

For peer review only

# Vaccine hesitancy and HPV vaccine uptake among male and female youth in Switzerland: a cross-sectional study

**Authors**: Laura M. Kiener [1,2] *, Corina L. Schwendener[1,2] * Kristen Jafflin [3,2], Audrey Meier[1,2], Noah Reber[1,2], Susanna Schärli[4], Franco Muggli[5], Nejla Gültekin[6], Benedikt M. Huber[7], Sonja Merten[3,2], Michael J. Deml [3,2], Philip E. Tarr [1,2]

*: equal contribution

**Affiliations:**

1. University Department of Medicine, Kantonsspital Baselland, University of Basel, 4101 Bruderholz, Switzerland
2. University of Basel, Petersplatz 1, 4051 Basel, Switzerland
3. Swiss Tropical and Public Health Institute, Socinstr. 57, 4051 Basel, Switzerland
4. Rekrutierungszentrum Aarau, Schweizer Armee, Aarau, Switzerland
5. Rekrutierungszentrum Monte Ceneri, Schweizer Armee, Monte Ceneri, Switzerland
6. Kompetenzzentrum für Militär- und Katastrophenmedizin, Eidgenössisches Departement für Verteidigung, Bevölkerungsschutz und Sport VBS Schweizer Armee, Ittigen, Switzerland
7. Department of Pediatrics, HFR Fribourg Cantonal Hospital, Fribourg, Switzerland

**Correspondence:** Prof. Dr. med. Philip E. Tarr, National Research Program NFP74 Vaccine Hesitancy, University Dept. of Medicine, Kantonsspital Baselland, University of Basel, 4101 Bruderholz, Switzerland, philip.tarr@unibas.ch, phone +41 (61) 436 2212, fax +41 (61) 436 3670

**Word count**

3086 (exclusive title page, authors' contribution statements, abstract, keywords, strengths and limitations of this study, acknowledgments, ethics approval)

**Key words:**

HPV vaccine, HPV under-immunization, Youth Attitudes about Vaccines Scale, Parental Attitudes about Childhood vaccination, youth, vaccine hesitancy

For peer review only

## Author Contributions

Ms. Kiener and Ms. Schwendener recruited participating youth, conducted interviews, carried out the analysis, drafted the initial manuscript, and reviewed and revised the manuscript.

Dr. Jafflin conceptualized and designed the study, designed the data collection instruments, coordinated and supervised data collection, carried out the analyses, and reviewed and revised the manuscript.

Ms. Meier and Mr. Reber recruited participating youth, conducted interviews, and reviewed and revised the manuscript.

Dr. Schärli, Dr. Muggli and Dr. Gültekin recruited participating youth, and reviewed and revised the manuscript.

Dr. Huber conceptualized and designed the study, recruited participating providers, and reviewed and revised the manuscript.

Dr. Merten conceptualized and designed the study, designed the data collection instruments, coordinated and supervised data collection, and reviewed and revised the manuscript.

Dr. Deml conceptualized and designed the study, designed the data collection instruments, and reviewed and revised the manuscript.

Dr. Tarr conceptualized and designed the study, designed the data collection instruments, recruited participating providers, coordinated and supervised data collection, carried out the analyses, and reviewed and revised the manuscript.

All authors approved the final manuscript as submitted and agree to be accountable for all aspects of the work. All authors attest they meet the ICMJE criteria for authorship.

For peer review only

## Abstract

**Objectives** Identifying factors associated with HPV vaccine uptake is essential for designing successful vaccination programs. We aimed to examine the association between vaccine hesitancy (VH) and HPV vaccine uptake among male and female youth in Switzerland.

**Design** With a cross-sectional study, an interview-based questionnaire was used to collect information on sociodemographic factors, vaccination records, and to measure the prevalence of VH using the Youth Attitudes about Vaccines scale (YAV-5), a modified version of the Parent Attitudes about Childhood Vaccinations (PACV) survey instrument.

**Setting and Participants** Eligible male and female participants, 15-26 years of age, were recruited through physicians' offices and military enlistment in all three language regions of Switzerland. Of 1001 participants, we included 674 participants with a vaccination record available (415 males, 259 females) in this study.

**Primary and secondary outcome measures** The outcome was uptake for HPV vaccine (having received ≥1 dose of HPV vaccine). Co-variates were VH, sex, age and other sociodemographics.

**Results** 151 (58%) female and 64 (15%) male participants received ≥1 dose of HPV vaccine. 81 (31%) female and 92 (22%) male participants were VH (YAV-5-Score ≥50). The odds for being unvaccinated were higher for VH females than non-VH females, adjusted odds ratio (OR)=4.90 (95% CI, 2.53-9.50), but similar among VH and non-VH males, OR=1.90 (95% CI, 0.84-4.31). The odds for being unvaccinated were lower for younger males (born on or after July 1, 2002) than older males (born before July 1, 2002), OR=0.34 (95% CI, 0.14-0.81), but we found no association between age and vaccine uptake for female youth, OR=0.97 (95% CI, 0.48-1.97).

**Conclusions** VH was associated with lower HPV vaccine uptake in female youth but not male youth in our study sample in Switzerland. Our findings suggest that issues other than VH contribute to HPV under-immunization in male youth in Switzerland.


For peer review only

## Strengths and limitations of this study:

- This study is the first using the YAV-5 to measure VH in youth and to associate VH with HPV vaccination uptake.

- The sample includes females and a large number of male youth from all three language regions of Switzerland, which allowed us to gain important insight into males' perspectives and vaccine uptake.

- The findings of this study are likely comparable to other high-income countries in which HPV vaccination programs have recently been extended to young males and where HPV vaccine is covered by health insurance or state-funded programs.

- The large age range of the participants might diminish effects of HPV vaccine recommendations and programs since programs have changed frequently in the past years.

- As our participant sampling is not representative, VH participants might be less likely to participate in a study examining VH than non-VH persons.

For peer review only


For peer review only - http://bmjopen.bmj.com/site/about/guidelines.xhtml

# 1. Introduction

Identifying factors contributing to under-immunization is essential for designing successful human papillomavirus (HPV) vaccination programs. Previous studies have shown various influences on HPV under-immunization in female youth, including access barriers, lack of efficient school vaccination programs, and concerns about safety and side effects [1-9]. Because many countries have only recently recommended HPV vaccine for males, information on the determinants of HPV vaccine uptake in men is limited, with lack of awareness and knowledge, access barriers, and lack of health care provider recommendation being previously reported determinants [4-6 10-17].

Vaccine hesitancy (VH), which "refers to delay in acceptance or refusal of vaccination despite availability of vaccination services" [18] was identified by the World Health Organization as among 10 threats to global health in 2019 [19]. Parental VH contributes to under-immunization of their children for both childhood vaccines [20-22] and HPV vaccine [23 24].

Since the determinants of HPV vaccination are context specific, local studies are needed. Additionally, previous studies investigating VH towards HPV vaccine had a limited sample size [24] or only investigated parents' perspectives without taking into account youth perspectives [13 17 23 25-28]. Exploring parents' perspectives is appropriate in the target group for HPV vaccine, i.e. adolescents 11-14 years of age, ideally before starting sexual activity. However, many countries, including Switzerland, allow adolescents as young as 14 to make vaccine decisions [29]. In addition, HPV vaccine is now recommended as a catch-up vaccine until 26 years in many countries, and until age 45 in the US [30]. Therefore, youth and young adult attitudes and perspectives on HPV vaccine, including VH, merit further investigation on a larger scale. This study addresses these research gaps by examining youth's VH in relationship to their HPV vaccination uptake.

The Swiss Federal Office of Public Health (FOPH) and the Federal Vaccination Commission have recommended HPV vaccine since 2007 as a routine vaccine for girls 11 to 14 years of age and young women as catch-up vaccination 15 to 26 years of age [31]. In 2015, the FOPH



For peer review only - http://bmjopen.bmj.com/site/about/guidelines.xhtml

added HPV vaccine to the list of optional vaccines for boys and young men with the same age groups as for females [32]. HPV vaccine is a key vaccine included in the Swiss National Vaccination Strategy [33], and cantonal (state) vaccination programs fully cover the cost of HPV vaccination for both males and females in the recommended age groups. Nevertheless, HPV vaccination coverage in Switzerland remains low [34 35]. While vaccine uptake has increased in the last decade [2], in the most recent assessment by the FOPH (period 2017-2019), only 64% and 20% of 16-year old girls and boys, respectively, had received $\geq$1 dose of HPV vaccine [34]. Regional differences in uptake have been attributed to socio-cultural factors, differences in vaccination policies, and differences in the strategies and efforts of the local health departments [36 37].

We use the Youth Attitudes about Vaccines Scale (YAV-5), a modified version of the Parent Attitudes about Childhood Vaccination (PACV) tool, originally developed by Opel and colleagues, in order to measure youth VH [38]. The PACV is a validated measure of VH predicting under-immunization in children in the US [20]. However, researchers found no association between PACV score and under-immunization with adolescent vaccines, including HPV, in the US [39]. With the later introduction of the HPV vaccine recommendation for males, we expect factors other than VH, such as HPV vaccine awareness, to influence uptake in males. The objectives of this study are to particularly assess how VH influences HPV vaccine uptake among males and females 15-26 years old in our sample in Switzerland. We hypothesize that (1) a YAV-5-score $\geq$50 (indicating VH) in youth is associated with lower HPV vaccine uptake, and (2) the association between YAV-5- score and HPV vaccine uptake is stronger among females than among males.



For peer review only - http://bmjopen.bmj.com/site/about/guidelines.xhtml

## 2. Methods

We conducted this cross-sectional study in the context of the Swiss National Research Program NRP74 on the determinants of VH and under-immunization with HPV and childhood vaccines in Switzerland [40]. The local ethics committee (Ethikkommission Nordwest- und Zentralschweiz, EKNZ; project ID number 2017-00725) approved the study, and ethics approval covers all study participants, including adolescent and adult patients, as well as biomedical and CAM providers. All participants provided informed consent after the nature and possible consequences of the study had been fully explained. Full details on the study, including recruitment methods, power calculation, development of the survey and a description of the situation of biomedicine and complementary and alternative (CAM) medicine in Switzerland have been previously published [41].

**2.1 Sampling.** Participants were male and female youth 15-26 years of age. We recruited participants in the offices of physicians providing either conventional biomedicine alone ("biomedical provider") or biomedicine combined with complementary and alternative medicine ("CAM provider"). We recruited participants from January 2019 to May 2020 in urban and rural areas in the three main language regions of Switzerland (German, French and Italian). In order to supplement the low number of male participants, we also recruited study participants during their military enlistment in the Swiss Army. Military service is mandatory for all Swiss men, with enlistment taking place at around 19 years of age. Participants recruited in physicians' offices were interviewed on the phone after the physician visit. Participants recruited through the military were interviewed face-to-face on site. Medical students trained by senior researchers in recruiting and obtaining informed consent conducted the interviews, which lasted 25-35 minutes. Data collectors entered all data into open data kit (ODK) using tablets.

**2.2 YAV-5.** We measure vaccine hesitancy using the Youth attitudes about Vaccines scale (YAV-5), an adapted version of the PACV survey instrument, which was originally developed by Opel and colleagues [38]. We have previously validated a 5-item short version of the PACV



For peer review only - http://bmjopen.bmj.com/site/about/guidelines.xhtml

For peer review only

in German, French, Italian and English regarding childhood vaccines [42]. For this project we adapted the questions for use in youth, and validated the YAV-5 in German, French, Italian and English language [43]. The adaptions we made for the YAV-5 instrument involved asking youth about their attitudes about adolescent vaccines. Each YAV-5-item is scored from 0-2. Individual scores are then summed and transformed to a 0-100 scale using simple linear transformation [20 44]. We will refer to participants with a score <50 as non-VH, and participants with a score ≥50 as VH [20 45].

**2.3 Survey.** In addition to the YAV-5, the survey included items about participants' socio-demographic characteristics such as language and birthdates. After finalizing the survey in English, we translated it into German, French and Italian using the forward and backward method [41], pre-tested, adjusted and piloted the survey as previously described [42]. We determine youths' HPV vaccination status through an examination of copies of their vaccination record and, for those not having a vaccination record because of they haven't received any vaccines, a personal statement to this effect. Being unvaccinated was defined as not having received ≥1 dose of HPV vaccine at the time of the interview.

**2.4 Participant age groups**. We divided participants into 2 age groups based on the later introduction of the recommendation of the HPV vaccine for boys in Switzerland. HPV vaccine has been covered by health insurance for male adolescents 11 to 14 years of age and as a catch-up vaccination until the age of 26 years since July 1, 2016. We therefore refer to male participants who were 14 years of age or older when insurance coverage for HPV vaccination began (born before July 1, 2002) as "older" and those who were under 14 at this time (born on or after July 1, 2002) as "younger" participants, respectively. For comparison, we applied the same age cut-offs to female participants.

**2.6 Patient and public involvement**

This study did not include patient or public involvement in designing the study, commenting the outcomes, interpreting the results or reviewing this manuscript.



For peer review only - http://bmjopen.bmj.com/site/about/guidelines.xhtml

**2.7 Quantitative data analysis.** We present descriptive statistics to summarize participants' age, place of birth, language region, YAV-5 score, and their HPV vaccination status. We calculated unadjusted odds ratios for being unvaccinated for all independent variables. We then conducted multi-level logistic regressions with random effects by recruitment setting (military enlistment, biomedical office, CAM office) to calculate adjusted odds ratios. Due to the high prevalence of vaccine hesitancy, calculated odds ratios are significantly higher than prevalence ratios would be, however, as these models are standard in the literature and allow easier adjustment for sampling frames, we use them here. Control variables included younger vs. older age group, being Swiss born, language region, and living in an area with local HPV vaccine school programs (Model 0). Next, we extended the model by adding the YAV-5-score as a new control variable for testing our hypotheses (Model 1). We also stratified analyses by sex. Additionally, we also checked for an interaction between vaccine hesitancy and age, but found none. Results of those analyses are available on request. We analyzed all data using STATA (v12.0, Stata Corp, College Station, TX).

For peer review only

# 3. Results

**3.1 Participants**. We completed interviews with 1001 youth. For our analysis, we exclude youth who did not match our aging criteria (n=15), who did not provide a correct postcode (n=10), or who did not provide vaccination records for any reason other than not having one due to not vaccinating (n=302).

All analyses are therefore based on 674 participants, of which 415 (62%) are male and 259 (38%) are female. Their characteristics are shown in **Table 1**. The majority of participants were Swiss born, spoke German and lived in the German-speaking region of Switzerland, consistent with this being the largest Swiss language region. We recruited the majority of male participants during military enlistment and the majority of female participants in biomedical physicians' offices. We recruited more female than male participants in CAM physicians' offices. 151 (58%) female and 64 (15%) male participants had received ≥1 dose of HPV vaccine. 81 (31%) females and 92 (22%) males were VH with a YAV-5-score ≥50.

**3.2. Determinants of HPV vaccine uptake, entire study population**. In univariable analysis, the odds of being unvaccinated with HPV were higher for males than for females (odds ratio [OR]=5.28; 95% confidence interval [CI], 3.17-8.80), and higher among participants recruited during military enlistment, most of whom were male, and in CAM offices (**Table 2**). VH youth had higher odds of being unvaccinated than non-VH youth, OR=2.75 (95% CI, 1.69-4.48). We found no significant difference in the odds of being unvaccinated in the univariable analysis based on age, being Swiss-born, language region or living in areas with local school vaccination programs. In the multivariable model 0, the odds were similar to the odds calculated in univariable analysis, with the exception of Swiss-born participants, whose odds for being unvaccinated were higher than foreign-born participants' (**Table 2**). In the multivariable model 1, after adding VH, the odds remained essentially unchanged: as in univariable analysis, the odds of being unvaccinated were higher among VH participants than non-VH participants, OR=3.20 (95% CI, 1.94-5.26).



For peer review only - http://bmjopen.bmj.com/site/about/guidelines.xhtml

**3.3. Determinants of HPV vaccine uptake, stratified by sex**. The univariable odds for being unvaccinated were lower for younger males compared to older males, but we found no significant difference in the odds among younger and older females (**Table 3**). The univariable odds for being unvaccinated were higher for females recruited in CAM offices than for females recruited in biomedical offices, and the odds for being unvaccinated were higher in VH females than non-VH females, OR=5.29 (95% CI, 2.71-10.33). We found no correlation between recruitment setting or VH and vaccine uptake for males (**Table 3**). There was no significant association between place of birth, language region, and school vaccination programs and vaccine uptake. In the multivariable model 0 and model 1, results for both males and females were similar to univariable results (**Table 3**). The odds for being unvaccinated were higher among VH females than non-VH females, OR=4.90 (95% CI, 2.53-9.50), but we found no association between VH and vaccine uptake for males, OR=1.90 (95% CI, 0.84-4.31). Similar, the odds for being unvaccinated were higher among females recruited in a CAM office than females recruited in a biomedical office even when controlling for VH in model 1, OR=2.52 (95%CI, 1.13-5.63), but for males we found no association between recruitment setting and vaccine uptake, OR=1.43 (95% CI, 0.39-5.20).


For peer review only - http://bmjopen.bmj.com/site/about/guidelines.xhtml

## 4. Discussion

This study shows that male youth have higher odds of being unvaccinated against HPV than females, despite fewer males being VH in our study. We found a significant association between VH and HPV under-immunization only for females. The main finding of the present study shows that VH is a major determinant of HPV under-immunization for female youth but not for male.

HPV vaccine uptake in our study was far lower in males than in females, despite the HPV vaccine recommendation having been officially made >4 years prior to the interviews. Our finding of higher HPV vaccine uptake in younger male participants than older ones suggests that the Swiss HPV vaccine recommendation for boys in 2015 led to a higher HPV vaccine uptake among younger eligible males. The finding of lower HPV vaccine uptake among older male youth point suggests a need for public health campaigns designed to increase awareness of catch-up vaccinations for male youth.

The VH prevalence in our study was almost 10 percentage points lower in males than in females, but this difference might be influenced by the fact that female participants were selected in a less random sample compared to males, who mostly were recruited through military enlistment. Still, this finding is consistent with previous reports suggesting that in general women tend to be more VH than men [46]. However, as suggested by the WHO's SAGE (Strategic Advisory Group of Experts on Immunization) working group on VH, a high level of VH leads to a lower vaccine demand, but lower VH levels do not necessarily lead to higher vaccine demand [18].

The finding that VH is not significantly associated with HPV under-immunization in males suggests that male youth face additional barriers to HPV vaccination, which may include limited HPV vaccine awareness and knowledge, access barriers, and a lack of priority given to male HPV vaccination by public health authorities and providers. An explanation for higher access to the HPV vaccine among female youth is that catch-up HPV vaccination in young Swiss women is partially done by gynecologists [35]. In contrast, young men in good health see



For peer review only - http://bmjopen.bmj.com/site/about/guidelines.xhtml

a medical provider less often than young women in good health [47] and may therefore not receive an HPV vaccine recommendation from any provider [4 5], nor be aware of the benefits of HPV vaccine [4 5 11-14].

To our knowledge, this is the first study that measures VH in youth using the YAV-5 instrument, an adapted version of the PACV survey, in order to examine associations between VH and HPV vaccine uptake. Similarly, we here show a significant association of youth VH and HPV under-immunization in female youth using the YAV-5-score. Our results echo the findings of Preston and colleagues who identified an association between negative attitudes toward HPV vaccine and HPV under-immunization among female, but not among male college students in Florida, USA [24]. We also found that females recruited in the offices of CAM providers have higher odds of being unvaccinated with HPV vaccine, even when controlling for youth vaccine hesitancy. This is consistent with previous research showing that CAM usage is associated with under-immunization [48 49].



For peer review only - http://bmjopen.bmj.com/site/about/guidelines.xhtml

## 5. Strengths and limitations

Strengths of our research include being the first study using the YAV-5 to measure VH in youth and to associate VH with HPV vaccination uptake. Additional strengths of our study include integration of a large number of male youth from all three language regions of Switzerland, which allowed us to gain important insight into males' perspectives and vaccine uptake. Little research has been done on males' HPV vaccine attitudes and uptake, particularly in the Swiss setting.

Our findings are likely comparable to other high-income countries in which HPV vaccination programs have recently been extended to young males and where HPV vaccine is covered by health insurance. Since the YAV-5 survey instrument has been previously validated to measure VH in youth [43], our study results can lead to meaningful comparisons with other populations.

Our study also has limitations. There was no evidence in our study that the availability of an HPV school vaccination program was associated with increased HPV vaccination uptake. This stands in contrast to a 2018 Swiss study on the association between school vaccination programs and HPV vaccine uptake in young women [2]. However, our study has important limitations for investigating the impact of school programs due to the large age range of participants, a non-representative sample, lack of data on schooling received, and the possibility that youth no longer live in the same area as when they would have received such schooling.

Our sample is not representative for Switzerland. Additional potential sources of bias result from us not being able to include participants who do not go to a physicians' office. This bias applies more to females in our study and less to males because the majority of males were recruited during military enlistment. Finally, VH persons might be less likely to participate in a study examining VH than non-VH persons. Future research could address these gaps by using a random sampling approach throughout the population with the aims of gathering representative data.



For peer review only - http://bmjopen.bmj.com/site/about/guidelines.xhtml

For peer review only

## 6. Conclusion

By administering the YAV-5, a validated instrument for measuring VH in youth [43], to a large sample of youth in Switzerland, our results suggest that VH may be a major determinant of HPV under-immunization for female youth. While male youth had higher odds of being unvaccinated, VH was not associated with HPV under-immunization in male youth. This suggests that issues other than VH, including HPV vaccine awareness and knowledge and access to HPV vaccination for youth who do not have regular contacts with the healthcare system, may still be predominant reasons for low vaccine uptake among male youth in Switzerland and should be explored in further research. To increase HPV vaccine uptake in Switzerland among both male and female youth, improving the design of HPV vaccination programs is essential. Future vaccination programs should address both female and male youth by not only increasing awareness and knowledge around HPV but also by optimizing access to HPV vaccines. This is a key issue for many eligible and generally healthy youth who do not have regular contacts with the healthcare system. Such efforts could be implemented through strengthening HPV vaccination in school programs for adolescents and by efforts aimed at raising awareness and improving access for catch-up vaccinations.

## Acknowledgements

We would like to thank the participating adolescents, young adults and physicians and their medical assistants for their time and effort.

## Disclosure of Potential Conflict of Interest

All authors: no conflicts.


For peer review only - http://bmjopen.bmj.com/site/about/guidelines.xhtml

## Funding

The study is funded via the Swiss National Science Foundation (Grant Number 407440_167398, recipient: PET), in the setting of National Research Programme (NRP) 74. The study benefited from supplementary postdoctoral fellowship funding from the Nora van Meeuwen-Haefliger-Foundation. No funding was obtained from vaccine manufacturers or the Swiss Federal Office of Public Health.

For peer review only

## Ethics approval

The local ethics committee (Ethikkommission Nordwest- und Zentralschweiz, EKNZ; project ID number 2017-00725) approved the study, and ethics approval covers all study participants, including adolescent and adult patients, as well as biomedical and CAM providers. All participants provided informed consent after the nature and possible consequences of the study had been fully explained.

## Data sharing statement

Data are available upon reasonable request to the corresponding author.

## References

1. Lang P, Sinniger P, Wymann MN, et al. Evolution of the HPV vaccination coverage in Switzerland, 2008-2016. Swiss public health conference. Basel. Available from: https://organizerscongress.org/frontend/index.php?page_id=4147&additions_conferenceschedule_action=detail&additions_conferenceschedule_controller=paperList&pid=4441&hash=925078cf906fe3575968a0d451fd6a1e106903fb18506bfe44d7fe13e74b74b1.
2. Riesen M, Konstantinoudis G, Lang P, et al. Exploring variation in human papillomavirus vaccination uptake in Switzerland: a multilevel spatial analysis of a national vaccination coverage survey. *BMJ Open* 2018;8(5):e021006. doi: 10.1136/bmjopen-2017-021006 [published Online First: 2018/05/19]
3. Wymann MN, Zographos AS, Altpeter E, et al. Human papillomavirus vaccine uptake in adolescence and adherence to cervical cancer screening in Switzerland: a national cross-


For peer review only - http://bmjopen.bmj.com/site/about/guidelines.xhtml

sectional survey. *Int J Public Health* 2018;63(1):105-14. doi: 10.1007/s00038-017-1050-x [published Online First: 2017/11/08]

4. Thompson EL, Rosen BL, Vamos CA, et al. Human Papillomavirus Vaccination: What Are the Reasons for Nonvaccination Among U.S. Adolescents? *J Adolesc Health* 2017;61(3):288-93. doi: 10.1016/j.jadohealth.2017.05.015 [published Online First: 2017/08/27]
5. Gilkey MB, Moss JL, McRee AL, et al. Do correlates of HPV vaccine initiation differ between adolescent boys and girls? *Vaccine* 2012;30(41):5928-34. doi: 10.1016/j.vaccine.2012.07.045 [published Online First: 2012/07/31]
6. Rodriguez SA, Mullen PD, Lopez DM, et al. Factors associated with adolescent HPV vaccination in the U.S.: A systematic review of reviews and multilevel framework to inform intervention development. *Prev Med* 2020;131:105968. doi: 10.1016/j.ypmed.2019.105968 [published Online First: 2019/12/28]
7. Canfell K, Egger S, Velentzis LS, et al. Factors related to vaccine uptake by young adult women in the catch-up phase of the National HPV Vaccination Program in Australia: Results from an observational study. *Vaccine* 2015;33(20):2387-94. doi: 10.1016/j.vaccine.2015.01.024 [published Online First: 2015/04/07]
8. Amadane M, de Pree C, Viviano M, et al. Characteristics of HPV-unvaccinated undergraduate health students in Switzerland, a cross sectional study. *Arch Public Health* 2019;77:29. doi: 10.1186/s13690-019-0348-y [published Online First: 2019/06/27]
9. Barrense-Dias Y., Akre C., Berchtold A., et al. Sexual health and behavior of young people in Switzerland. *Raison de santeé* 2018;291 doi: http://dx.doi.org/10.16908/issn.1660-7104/291
10. Sherman SM, Nailer E. Attitudes towards and knowledge about Human Papillomavirus (HPV) and the HPV vaccination in parents of teenage boys in the UK. *PLoS One* 2018;13(4):e0195801. doi: 10.1371/journal.pone.0195801 [published Online First: 2018/04/12]
11. Cooper DL, Zellner-Lawrence T, Mubasher M, et al. Examining HPV Awareness, Sexual Behavior, and Intent to Receive the HPV Vaccine Among Racial/Ethnic Male College Students 18-27 years. *Am J Mens Health* 2018;12(6):1966-75. doi: 10.1177/1557988318803163 [published Online First: 2018/10/20]
12. Perez S, Tatar O, Gilca V, et al. Untangling the psychosocial predictors of HPV vaccination decision-making among parents of boys. *Vaccine* 2017;35(36):4713-21. doi: 10.1016/j.vaccine.2017.07.043 [published Online First: 2017/08/02]
13. Lacombe-Duncan A, Newman PA, Baiden P. Human papillomavirus vaccine acceptability and decision-making among adolescent boys and parents: A meta-ethnography of qualitative studies. *Vaccine* 2018;36(19):2545-58. doi: 10.1016/j.vaccine.2018.02.079 [published Online First: 2018/04/08]
14. Kasymova S. Human papillomavirus (HPV) and HPV vaccine knowledge, the intention to vaccinate, and HPV vaccination uptake among male college students. *J Am Coll Health* 2020:1-15. doi: 10.1080/07448481.2020.1785471 [published Online First: 2020/07/17]
15. Voss DS, Wofford LG. Human Papillomavirus Vaccine Uptake in Adolescent Boys: An Evidence Review. *Worldviews Evid Based Nurs* 2016;13(5):390-95. doi: 10.1111/wvn.12172 [published Online First: 2016/07/28]
16. Dibble KE, Maksut JL, Siembida EJ, et al. A Systematic Literature Review of HPV Vaccination Barriers Among Adolescent and Young Adult Males. *J Adolesc Young Adult Oncol* 2019;8(5):495-511. doi: 10.1089/jayao.2019.0004 [published Online First: 2019/05/16]
17. Radisic G, Chapman J, Flight I, et al. Factors associated with parents' attitudes to the HPV vaccination of their adolescent sons : A systematic review. *Prev Med* 2017;95:26-37. doi: 10.1016/j.ypmed.2016.11.019 [published Online First: 2016/12/10]
18. MacDonald NE. Vaccine hesitancy: Definition, scope and determinants. *Vaccine* 2015;33(34):4161-4. doi: 10.1016/j.vaccine.2015.04.036 [published Online First: 2015/04/22]
19. World health organization. Ten threats to global health in 2019 2019 [Available from: https://www.who.int/news-room/spotlight/ten-threats-to-global-health-in-2019 accessed 11.02.2021.



For peer review only - http://bmjopen.bmj.com/site/about/guidelines.xhtml

20. Opel DJ, Taylor JA, Zhou C, et al. The relationship between parent attitudes about childhood vaccines survey scores and future child immunization status: a validation study. *JAMA Pediatr* 2013;167(11):1065-71. doi: 10.1001/jamapediatrics.2013.2483 [published Online First: 2013/09/26]
21. Williams SE, Morgan A, Opel D, et al. Screening Tool Predicts Future Underimmunization Among a Pediatric Practice in Tennessee. *Clin Pediatr (Phila)* 2016;55(6):537-42. doi: 10.1177/0009922815615823 [published Online First: 2015/11/20]
22. Napolitano F, D'Alessandro A, Angelillo IF. Investigating Italian parents' vaccine hesitancy: A cross-sectional survey. *Hum Vaccin Immunother* 2018;14(7):1558-65. doi: 10.1080/21645515.2018.1463943 [published Online First: 2018/04/12]
23. Szilagyi PG, Albertin CS, Gurfinkel D, et al. Prevalence and characteristics of HPV vaccine hesitancy among parents of adolescents across the US. *Vaccine* 2020;38(38):6027-37. doi: 10.1016/j.vaccine.2020.06.074 [published Online First: 2020/08/08]
24. Preston SM, Darrow WW. Are Men Being Left Behind (Or Catching Up)? Differences in HPV Awareness, Knowledge, and Attitudes Between Diverse College Men and Women. *Am J Mens Health* 2019;13(6):1557988319883776. doi: 10.1177/1557988319883776 [published Online First: 2019/12/04]
25. Della Polla G, Pelullo CP, Napolitano F, et al. HPV vaccine hesitancy among parents in Italy: a cross-sectional study. *Hum Vaccin Immunother* 2020;16(11):2744-51. doi: 10.1080/21645515.2020.1744367 [published Online First: 2020/04/17]
26. Sherman SM, Bartholomew K, Denison HJ, et al. Knowledge, attitudes and awareness of the human papillomavirus among health professionals in New Zealand. *PLoS One* 2018;13(12):e0197648. doi: 10.1371/journal.pone.0197648 [published Online First: 2019/01/01]
27. Perez S, Tatar O, Shapiro GK, et al. Psychosocial determinants of parental human papillomavirus (HPV) vaccine decision-making for sons: Methodological challenges and initial results of a pan-Canadian longitudinal study. *BMC Public Health* 2016;16(1):1223. doi: 10.1186/s12889-016-3828-9 [published Online First: 2016/12/07]
28. Newman PA, Logie CH, Lacombe-Duncan A, et al. Parents' uptake of human papillomavirus vaccines for their children: a systematic review and meta-analysis of observational studies. *BMJ Open* 2018;8(4):e019206. doi: 10.1136/bmjopen-2017-019206 [published Online First: 2018/04/22]
29. Yang YT, Olick RS, Shaw J. Adolescent Consent to Vaccination in the Age of Vaccine-Hesitant Parents. *JAMA Pediatr* 2019;173:1119-216. doi: 10.1001/jamapediatrics.2019.3330 [published Online First: 2019/10/08]
30. Meites E, Szilagyi PG, Chesson HW, et al. Human Papillomavirus Vaccination for Adults: Updated Recommendations of the Advisory Committee on Immunization Practices. *MMWR Morb Mortal Wkly Rep* 2019;68(32):698-702. doi: 10.15585/mmwr.mm6832a3 [published Online First: 2019/08/16]
31. Swiss Federal office of public health. Recommandations de vaccination contre les papillomavirus humains (HPV), 2008. Document in French: https://www.bag.admin.ch/dam/bag/fr/dokumente/mt/i-und-b/richtlinienempfehlungen/empfehlungen-spezifische-erreger-krankheiten/hpv/hpv-humanepapillomaviren-empfehlung-impfung.pdf.download.pdf/ofsp-recommandations-hpv.pdf
32. Swiss Federal office of public health. Vaccination contre les HPV : recommandation de vaccination complémentaire pour les garçons et jeunes hommes âgés de 11 à 26 ans. BAG Bulletin, 2015:141-50. Document in French: https://www.bag.admin.ch/dam/bag/fr/dokumente/mt/i-und-b/richtlinien-empfehlungen/empfehlungen-spezifische-erreger-krankheiten/hpv/hpv-impfung-jungenmaenner.pdf.download.pdf/hpv-vaccination-garcons.pdf
33. Swiss Federal office of public health. Stratégie nationale de vaccination, 2017. Document in French: https://www.bag.admin.ch/dam/bag/fr/dokumente/mt/i-und-i/nsi/nsi-strategievollversion.

For peer review only

pdf.download.pdf/nsi-strategie-vollversion-fr.pdf
34. Swiss Federal office of public health. Couverture vaccinale des enfants âgés de 2, 8 et 16 ans en Suisse, 1999-2019 2020 [updated 30.07.2020. Document in French: https://www.bag.admin.ch/dam/bag/fr/dokumente/mt/i-und-b/durchimpfung/tabelledurchimpfung.xlsx.download.xlsx/tabelle-durchimpfung-200730-fr.xlsx.
35. Swiss Federal office of public health. La vaccination contre le HPV en Suisse: résultats d'une enquête nationale réalisée en 2014. BAG Bulletin, 2015:445-52. Document in French: https://www.bag.admin.ch/dam/bag/fr/dokumente/cc/Kampagnen/Bulletin/2015/BU_15_15_f.pdf.download.pdf/BU_15_15_f.pdf
36. Masserey Spicher V, Weiss MG. Policy and socio-cultural differences between cantons in Switzerland with high and low adolescent vaccination coverage for hepatitis B and HPV. *Vaccine* 2019;37(52):7539-46. doi: 10.1016/j.vaccine.2019.09.085 [published Online First: 2019/10/22]
37. Riesen M, Garcia V, Low N, et al. Modeling the consequences of regional heterogeneity in human papillomavirus (HPV) vaccination uptake on transmission in Switzerland. *Vaccine* 2017;35(52):7312-21. doi: 10.1016/j.vaccine.2017.10.103 [published Online First: 2017/11/12]
38. Opel DJ, Mangione-Smith R, Taylor JA, et al. Development of a survey to identify vaccine-hesitant parents: the parent attitudes about childhood vaccines survey. *Hum Vaccin* 2011;7(4):419-25. doi: 10.4161/hv.7.4.14120 [published Online First: 2011/03/11]
39. Roberts JR, Thompson D, Rogacki B, et al. Vaccine hesitancy among parents of adolescents and its association with vaccine uptake. *Vaccine* 2015;33(14):1748-55. doi: 10.1016/j.vaccine.2015.01.068 [published Online First: 2015/02/11]
40. National Research Programme 74. 28 Vaccine-sceptical patients and doctors in Switzerland: National Research Programme 74; www.nfp74.ch/en/projects/out-patient-care/project-tarr accessed 14.02.2021.
41. Deml MJ, Jafflin K, Merten S, et al. Determinants of vaccine hesitancy in Switzerland: study protocol of a mixed-methods national research programme. *BMJ Open* 2019;9(11):e032218. doi: 10.1136/bmjopen-2019-032218 [published Online First: 2019/11/05]
42. Olarewaju VO, Jafflin K, Deml MJ, et al. Application of the Parent Attitudes about Childhood Vaccines (PACV) survey in three national languages in Switzerland: Exploratory factor analysis and Mokken scale analysis. *Hum Vaccin Immunother* 2021:1-9. doi: 10.1080/21645515.2021.1894894 [published Online First: 2021/03/25]
43. Olarewaju VO, Jafflin K, Deml MJ, et al. The Youth Attitudes about Vaccines (YAV-5) scale: adapting the parent attitudes about childhood vaccines short scale for use with youth in German, French, and Italian in Switzerland, exploratory factor analysis and mokken scaling analysis. *Hum Vaccin Immunother* 2021:1-8. doi: 10.1080/21645515.2021.1980314 [published Online First: 2021/11/10]
44. Oladejo O, Allen K, Amin A, et al. Comparative analysis of the Parent Attitudes about Childhood Vaccines (PACV) short scale and the five categories of vaccine acceptance identified by Gust et al. *Vaccine* 2016;34(41):4964-68. doi: 10.1016/j.vaccine.2016.08.046 [published Online First: 2016/08/28]
45. Opel DJ, Banerjee A, King P, et al. Washington State Licensed Child Care Facility Directors' Perspectives on Childhood Immunization. *J Pediatric Infect Dis Soc* 2013;2(1):40-9. doi: 10.1093/jpids/pis088 [published Online First: 2013/03/01]
46. Baumgaertner B, Ridenhour BJ, Justwan F, et al. Risk of disease and willingness to vaccinate in the United States: A population-based survey. *PLoS Med* 2020;17(10):e1003354. doi: 10.1371/journal.pmed.1003354 [published Online First: 2020/10/16]
47. Ashman JJ, Rui P, Okeyode T. Characteristics of Office-based Physician Visits, 2016. *NCHS Data Brief* 2019(331):1-8. [published Online First: 2019/02/02]
48. Zuzak TJ, Zuzak-Siegrist I, Rist L, et al. Attitudes towards vaccination: users of complementary and alternative medicine versus non-users. *Swiss Med Wkly* 2008;138(47-48):713-8. [published Online First: 2008/12/02]


For peer review only - http://bmjopen.bmj.com/site/about/guidelines.xhtml

49. Attwell K, Ward PR, Meyer SB, et al. "Do-it-yourself": Vaccine rejection and complementary and alternative medicine (CAM). *Soc Sci Med* 2018;196:106-14. doi: 10.1016/j.socscimed.2017.11.022 [published Online First: 2017/11/28]

For peer review only



For peer review only - http://bmjopen.bmj.com/site/about/guidelines.xhtml

**Table 1**. Participant characteristics for full sample and by sex

| | Total (n=674) n (%) | | Male (n=415) n (%) | | Female (n=259) n (%) | |
|---|---|---|---|---|---|---|
| **Age in years (median, IQR)** | 19.1 | (18.1, 20.7) | 19.2 | (18.4, 20.1) | 19.0 | (16.8, 21.7) |
| Born before July 1, 2002 | 563 | (83.5) | 370 | (89.2) | 193 | (74.5) |
| Born on or after July 1, 2002 | 111 | (16.5) | 45 | (10.8) | 66 | (25.5) |
| Swissborn | 621 | (92.1) | 382 | (92.1) | 239 | (92.3) |
| **Language region** | | | | | | |
| German | 494 | (73.3) | 321 | (77.4) | 173 | (66.8) |
| French | 43 | (6.4) | 16 | (3.9) | 27 | (10.4) |
| Italian | 137 | (20.3) | 78 | (18.8) | 59 | (22.8) |
| **Participant recruitment Setting** | | | | | | |
| Military enlistment | 287 | (42.6) | 283 | (68.2) | 4 | (1.5) |
| Biomedical provider | 280 | (41.5) | 97 | (23.4) | 183 | (70.7) |
| CAM provider | 107 | (15.9) | 35 | (8.4) | 72 | (27.8) |
| **HPV vaccine school program in area of their residence** | | | | | | |
| School program available | 366 | (54.3) | 232 | (55.9) | 134 | (51.7) |
| **Vaccination status** | | | | | | |
| Has received ≥ 1 dose of HPV vaccine | 215 | (31.9) | 64 | (15.4) | 151 | (58.3) |
| **YAV-5-Score** | | | | | | |
| Score ≥50 (hesitant) | 173 | (25.7) | 92 | (22.2) | 81 | (31.3) |
| YAV-5-Score mean (SD) | 32.3 | (23.4) | 30.4 | (22.3) | 35.3 | (24.8) |
| YAV-5-Score median (IQR) | 30.0 | (20, 50) | 30.0 | (10, 40) | 30.0 | (20, 50) |

Note: CAM, Complementary and alternative medicine; IQR, interquartile range; SD, standard deviation; YAV-5, Youth Attitudes about Vaccines Scale.

For peer review only

**Table 2**. Unadjusted and adjusted odds of not having received ≥1 dose of HPV vaccine, controlling for sex, age groups, place of birth, language region, recruitment setting, availability of HPV vaccine school program in the area of their residence, and VH.

| | **Univariable Analysis** | | | **Model 0** | | | **Model 1** | | |
|---|---|---|---|---|---|---|---|---|---|
| | OR | CI (95%) | | AOR | CI (95%) | | AOR | CI (95%) | |
| **Sex** | | | | | | | | | |
| Female | (reference) | | | (reference) | | | (reference) | | |
| Male | **5.28** | **(3.17** | **8.80)** | **5.16** | **(3.06** | **8.70)** | **5.77** | **(3.40** | **9.79)** |
| **Age groups based on birthdates** | | | | | | | | | |
| Born before July 1, 2002 | (reference) | | | (reference) | | | (reference) | | |
| Born on or after July 1, 2002 | 0.74 | (0.44 | 1.26) | 0.72 | (0.42 | 1.21) | 0.70 | (0.41 | 1.19) |
| **Place of birth** | | | | | | | | | |
| Outside Switzerland | (reference) | | | (reference) | | | (reference) | | |
| Switzerland | 1.85 | (0.95 | 3.60) | **2.03** | **(1.02** | **4.06)** | **2.05** | **(1.02** | **4.10)** |
| **Language region** | | | | | | | | | |
| German | (reference) | | | (reference) | | | (reference) | | |
| French | 0.79 | (0.33 | 1.89) | 0.89 | (0.39 | 2.04) | 0.87 | (0.38 | 1.98) |
| Italian | 0.54 | (0.26 | 1.12) | 0.57 | (0.29 | 1.13) | 0.60 | (0.31 | 1.17) |
| **Participant recruitment setting** | | | | | | | | | |
| Biomedical | (reference) | | | (reference) | | | (reference) | | |
| CAM | **2.46** | **(1.33** | **4.55)** | **2.29** | **(1.21** | **4.32)** | **2.09** | **(1.13** | **3.89)** |
| Military enlistment | **7.29** | **(2.64** | **20.09)** | 2.60 | (0.99 | 6.80) | **2.79** | **(1.15** | **6.79)** |
| **HPV vaccine school program in area of their residence** | | | | | | | | | |
| Not available | (reference) | | | (reference) | | | (reference) | | |
| Available | 1.37 | (0.85 | 2.21) | 1.26 | (0.72 | 2.19) | 1.20 | (0.69 | 2.08) |
| **YAV-5-Score** | | | | | | | | | |
| Score < 50 (not hesitant) | (reference) | | | | | | (reference) | | |
| Score ≥ 50 (hesitant) | **2.75** | **(1.69** | **4.48)** | | | | **3.20** | **(1.94** | **5.26)** |

Note: AOR, adjusted odds ratio; CAM, complementary and alternative medicine; CI, confidential interval; OR, odds ratio; YAV-5, Youth Attitudes about Vaccines Scale.

For peer review only

**Table 3**. Unadjusted and adjusted odds of not having received ≥1 dose of HPV vaccine stratified by sex; controlling for participant sex, age groups, place of birth, language region, recruitment setting, availability of HPV vaccine school program in the area of their residence and VH.

| | **Univariable Analysis, Male Only (n=415)** | | **Model 0, male only (n= 415)** | | **Model 1, male only (n=415)** | | **Univariable Analysis, Female Only (n=259)** | | **Model 0, female only (n=259)** | | **Model 1, female only (n=259)** | |
|---|---|---|---|---|---|---|---|---|---|---|---|---|
| | OR | CI (95%) | AOR | CI (95%) | AOR | CI (95%) | OR | CI (95%) | AOR | CI (95%) | AOR | CI (95%) |
| **Place of birth** | | | | | | | | | | | | |
| Outside Switzerland | (reference) | | (reference) | | (reference) | | (reference) | | (reference) | | (reference) | |
| Switzerland | 2.10 | (0.84 5.23) | 1.77 | (0.69 4.51) | 1.71 | (0.67 4.34) | 2.14 | (0.66 6.95) | 2.10 | (0.64 6.86) | 2.38 | (0.68 8.32) |
| **Age groups based on birthdates** | | | | | | | | | | | | |
| Born before July 1, 2002 | (reference) | | (reference) | | (reference) | | (reference) | | (reference) | | (reference) | |
| Born on or after July 1, 2002 | **0.29** | **(0.12 0.71)** | **0.34** | **(0.14 0.81)** | **0.31** | **(0.13 0.73)** | 0.94 | (0.46 1.93) | 0.97 | (0.48 1.97) | 1.14 | (0.56 2.33) |
| **Language region** | | | | | | | | | | | | |
| German | (reference) | | (reference) | | (reference) | | (reference) | | (reference) | | (reference) | |
| French | 0.50 | (0.11 2.33) | 0.72 | (0.16 3.20) | 0.69 | (0.16 3.03) | 0.88 | (0.31 2.52) | 0.99 | (0.34 2.90) | 0.94 | (0.32 2.80) |
| Italian | 0.38 | (0.12 1.21) | 0.57 | (0.18 1.82) | 0.57 | (0.18 1.80) | 0.44 | (0.17 1.12) | 0.56 | (0.20 1.58) | 0.59 | (0.22 1.62) |
| **Participant recruitment setting** | | | | | | | | | | | | |
| Biomedical | (reference) | | (reference) | | (reference) | | (reference) | | (reference) | | (reference) | |
| CAM | 2.63 | (0.66 10.45) | 1.59 | (0.44 5.82) | 1.43 | (0.39 5.20) | **3.08** | **(1.33 7.14)** | **2.75** | **(1.19 6.33)** | **2.52** | **(1.13 5.63)** |
| Military enlistment | 2.49 | (0.44 13.99) | 1.83 | (0.52 6.47) | 1.84 | (0.57 5.95) | 0.58 | (0.03 11.81) | 0.55 | (0.03 9.53) | 0.59 | (0.03 10.29) |
| **HPV vaccine school program in area of their residence** | | | | | | | | | | | | |
| Not available | (reference) | | (reference) | | (reference) | | (reference) | | (reference) | | (reference) | |
| Available | 1.39 | (0.67 2.91) | 1.21 | (0.53 2.76) | 1.20 | (0.53 2.72) | 1.74 | (0.88 3.43) | 1.14 | (0.48 2.71) | 1.00 | (0.42 2.37) |
| **YAV-5-Score** | | | | | | | | | | | | |
| Score < 50 (not hesitant) | (reference) | | | | (reference) | | (reference) | | | | (reference) | |
| Score ≥ 50 (hesitant) | 1.68 | (0.75 3.76) | | | 1.90 | (0.84 4.31) | **5.29** | **(2.71 10.33)** | | | **4.90** | **(2.53 9.50)** |

Note: AOR, adjusted odds ratio; CAM, complementary and alternative medicine; CI, confidential interval; OR, odds ratio; VH, vaccine hesitant; YAV-5, Youth Attitudes about Vaccines Scale.



For peer review only - http://bmjopen.bmj.com/site/about/guidelines.xhtml

**STROBE 2007 (v4) checklist of items to be included in reports of observational studies in epidemiology***
**Checklist for cohort, case-control, and cross-sectional studies (combined)**

| Section/Topic | Item # | Recommendation | Reported on page # |
|---|---|---|---|
| **Title and abstract** | 1 | (*a*) Indicate the study's design with a commonly used term in the title or the abstract | 1 |
| | | (*b*) Provide in the abstract an informative and balanced summary of what was done and what was found | 3 |
| **Introduction** | | | |
| Background/rationale | 2 | Explain the scientific background and rationale for the investigation being reported | 5,6 |
| Objectives | 3 | State specific objectives, including any pre-specified hypotheses | 6 |
| **Methods** | | | |
| Study design | 4 | Present key elements of study design early in the paper | 7 |
| Setting | 5 | Describe the setting, locations, and relevant dates, including periods of recruitment, exposure, follow-up, and data collection | 7 |
| Participants | 6 | (*a*) *Cohort study*—Give the eligibility criteria, and the sources and methods of selection of participants. Describe methods of follow-up<br>*Case-control study*—Give the eligibility criteria, and the sources and methods of case ascertainment and control selection. Give the rationale for the choice of cases and controls<br>*Cross-sectional study*—Give the eligibility criteria, and the sources and methods of selection of participants | 7 |
| | | (*b*) *Cohort study*—For matched studies, give matching criteria and number of exposed and unexposed<br>*Case-control study*—For matched studies, give matching criteria and the number of controls per case | - |
| Variables | 7 | Clearly define all outcomes, exposures, predictors, potential confounders, and effect modifiers. Give diagnostic criteria, if applicable | 7, 8 |
| Data sources/ measurement | 8* | For each variable of interest, give sources of data and details of methods of assessment (measurement). Describe comparability of assessment methods if there is more than one group | 7 |
| Bias | 9 | Describe any efforts to address potential sources of bias | 7 |
| Study size | 10 | Explain how the study size was arrived at | 7 |
| Quantitative variables | 11 | Explain how quantitative variables were handled in the analyses. If applicable, describe which groupings were chosen and why | 7 |
| Statistical methods | 12 | (*a*) Describe all statistical methods, including those used to control for confounding | 8,9 |
| | | (*b*) Describe any methods used to examine subgroups and interactions | 8,9 |
| | | (*c*) Explain how missing data were addressed | 10 |
| | | (*d*) *Cohort study*—If applicable, explain how loss to follow-up was addressed<br>*Case-control study*—If applicable, explain how matching of cases and controls was addressed | - |

For peer review only

| | | | |
|---|---|---|---|
| | | *Cross-sectional study*—If applicable, describe analytical methods taking account of sampling strategy | |
| | | (*e*) Describe any sensitivity analyses | - |
| **Results** | | | |
| Participants | 13* | (a) Report numbers of individuals at each stage of study—eg numbers potentially eligible, examined for eligibility, confirmed eligible, included in the study, completing follow-up, and analysed | 10 |
| | | (b) Give reasons for non-participation at each stage | 10 |
| | | (c) Consider use of a flow diagram | - |
| Descriptive data | 14* | (a) Give characteristics of study participants (eg demographic, clinical, social) and information on exposures and potential confounders | 10, 19 |
| | | (b) Indicate number of participants with missing data for each variable of interest | - |
| | | (c) *Cohort study*—Summarise follow-up time (eg, average and total amount) | - |
| Outcome data | 15* | *Cohort study*—Report numbers of outcome events or summary measures over time | - |
| | | *Case-control study*—Report numbers in each exposure category, or summary measures of exposure | - |
| | | *Cross-sectional study*—Report numbers of outcome events or summary measures | 10, 19 |
| Main results | 16 | (*a*) Give unadjusted estimates and, if applicable, confounder-adjusted estimates and their precision (eg, 95% confidence interval). Make clear which confounders were adjusted for and why they were included | 10, 11, 20, 21 |
| | | (*b*) Report category boundaries when continuous variables were categorized | - |
| | | (*c*) If relevant, consider translating estimates of relative risk into absolute risk for a meaningful time period | - |
| Other analyses | 17 | Report other analyses done—eg analyses of subgroups and interactions, and sensitivity analyses | - |
| **Discussion** | | | |
| Key results | 18 | Summarise key results with reference to study objectives | 12 |
| Limitations | 19 | Discuss limitations of the study, taking into account sources of potential bias or imprecision. Discuss both direction and magnitude of any potential bias | 14 |
| Interpretation | 20 | Give a cautious overall interpretation of results considering objectives, limitations, multiplicity of analyses, results from similar studies, and other relevant evidence | 12, 13, 15 |
| Generalisability | 21 | Discuss the generalisability (external validity) of the study results | 14 |
| **Other information** | | | |
| Funding | 22 | Give the source of funding and the role of the funders for the present study and, if applicable, for the original study on which the present article is based | 15 |

*Give information separately for cases and controls in case-control studies and, if applicable, for exposed and unexposed groups in cohort and cross-sectional studies.
**Note:** An Explanation and Elaboration article discusses each checklist item and gives methodological background and published examples of transparent reporting. The STROBE checklist is best used in conjunction with this article (freely available on the Web sites of PLoS Medicine at http://www.plosmedicine.org/, Annals of Internal Medicine at http://www.annals.org/, and Epidemiology at http://www.epidem.com/). Information on the STROBE Initiative is available at www.strobe-statement.org.

For peer review only
